# Survey on mathematical modeling of infectious disease dynamics: insights and applications

**DOI:** 10.1186/s12879-026-12905-7

**Published:** 2026-02-24

**Authors:** Neveen Ali Eshtewy, Ali Forootani, Zahra Ahangari Sisi

**Affiliations:** 1 Department of Mathematics, Faculty of Sciences, Arish University, Dahia Al-Salam, University Street, Arish, North Sinai, Egypt; 2 University of Nizwa, College of Arts and Sciences, Nizwa, Sultanate of Oman; 3 Max Planck Institute of Geoanthropology, Kahlaische Str. 10, 07745 Jena, Thüringen Germany; 4 Helmholtz Centre for Environmental Research – UFZ, Permoserstraße 15, 04318 Leipzig, Sachsen Germany; 5 Tabriz University of Technology – Sahand, Sahand, Tabriz, East Azerbaijan Province, Iran

**Keywords:** Mathematical modeling, Infectious diseases, Epidemiology

## Abstract

Mathematical modeling has become an indispensable tool for understanding, predicting, and controlling the spread of infectious diseases. Over the years, a wide variety of models have been developed to analyze disease dynamics and forecast epidemic trajectories. Deterministic and stochastic frameworks provide quantitative insights into transmission mechanisms and allow for rigorous evaluation of public health interventions such as quarantine, vaccination, and lockdown strategies. The integration of computational and data-driven methods has significantly advanced epidemic modeling. Techniques from network analysis, large-scale data processing, and artificial intelligence (AI) have improved both the accuracy and efficiency of model predictions. In particular, deep learning methods—most notably in medical imaging—enable fast and reliable automated diagnosis of disease. Moreover, the combination of mathematical modeling with AI facilitates real-time tracking and forecasting of outbreaks, thereby supporting public health authorities in optimizing resource allocation and ensuring timely responses. The increasing availability of open-source datasets, including case reports, demographic information, mobility patterns, and medical images, has further expanded the capabilities of data-driven epidemic models. Such approaches play a critical role in managing emerging infectious diseases, strengthening preparedness, and mitigating the societal impact of future outbreaks. This work provides a comprehensive overview of mathematical modeling approaches in infectious disease dynamics, emphasizing their relevance for public health emergency management and evidence-based intervention strategies.

## Introduction

Infectious diseases have posed profound challenges to humanity throughout history, shaping societies, economies, and demographic patterns across centuries. Major historical outbreaks—such as the bubonic plague in the fourteenth century and the cholera epidemics of the nineteenth century—left enduring marks on global health and collective memory. Cholera, transmitted through contaminated water and food, and tuberculosis, spread via the respiratory route, were among the most devastating diseases of their time. Despite significant advances in medicine and public health, tuberculosis remains a major global health concern, particularly in low- and middle-income regions, where it persists as one of the most critical re-emerging infectious diseases alongside malaria.

Emerging and re-emerging infectious diseases continue to threaten global health due to a combination of environmental change, human mobility, and social and behavioral factors. Diseases such as smallpox, the Spanish flu, dengue, and AIDS have repeatedly resurfaced—especially in tropical and underdeveloped areas—and remain among the leading causes of morbidity and mortality worldwide. These recurring epidemics underscore the urgent and ongoing need for adaptive, evidence-based, and resilient public health strategies to mitigate infectious disease transmission and to strengthen preparedness against future outbreaks [1, 2].

Mathematical modeling has played an essential role in advancing the understanding and control of infectious diseases. Its origins can be traced back to 1760, when Daniel Bernoulli developed the first mathematical model to assess the effectiveness of variolation, an early precursor to modern smallpox vaccination—marking a pivotal step in applying quantitative reasoning to disease prevention. During the nineteenth century, John Snow’s investigation of the cholera outbreak in London established him as a central figure in epidemiology, illustrating the power of systematic observation and data-driven analysis in identifying transmission pathways. Meanwhile, the pioneering work of Louis Pasteur, who confirmed that microorganisms cause many infectious diseases, and Edward Jenner’s development of the smallpox vaccine in 1796, provided crucial foundations for modern immunology and public health. The eventual eradication of smallpox in 1980, achieved through coordinated mass-vaccination programs, remains one of the greatest triumphs in disease control.

In modern public health, mathematical modeling has become an indispensable tool for examining the complex dynamics of infectious disease transmission. These models characterize interactions among pathogens, hosts, and environmental factors, offering critical insights into the mechanisms driving epidemics. They are also widely used to evaluate the effectiveness of interventions such as vaccination programs, quarantine measures, and antimicrobial therapies, as well as to simulate potential threats, including bioterrorism scenarios. Collectively, these scientific and mathematical advances form the foundation of the sophisticated prevention and control strategies used today.

Real-world events have demonstrated the practical value of these approaches. For example, predictive models played central roles during the 2001 foot-and-mouth disease outbreak in the United Kingdom and the 2003 SARS epidemic, informing public-health strategies, optimizing resource allocation, and helping to limit transmission. The continued integration of mathematical modeling with big-data analytics and artificial intelligence is expected to further strengthen infectious-disease management and enhance global health preparedness.

**Fig. 1:**
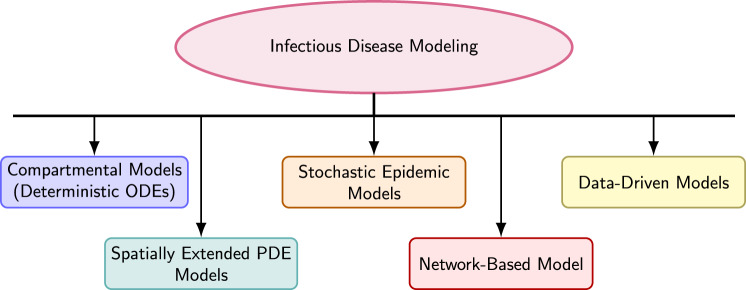
Hierarchical framework of infectious disease modeling approaches

In contrast to existing review articles that typically focus on a single modeling paradigm—such as compartmental frameworks [3], network-based approaches [4], or data assimilation and forecasting methods [5]—this survey adopts an integrative, cross-framework perspective that explicitly connects classical mechanistic models with emerging data-driven and artificial intelligence–based methods. Rather than presenting these approaches in isolation, the review emphasizes their complementary roles in epidemic analysis, forecasting, and policy support. By systematically organizing deterministic, stochastic, spatial, network, and AI-assisted models within a unified conceptual framework (Fig. 1), this work highlights how methodological advances in one domain can enhance performance and interpretability in others. This integrative viewpoint provides readers with a coherent roadmap for selecting and combining modeling strategies according to data availability, epidemic scale, and public health objectives.

Furthermore, this review advances beyond prior surveys by placing particular emphasis on translational relevance and practical deployment in public health decision-making. Alongside theoretical foundations, the manuscript synthesizes recent applications that demonstrate how hybrid modeling pipelines—combining mechanistic structure, large-scale data streams, and machine learning—enable real-time outbreak monitoring, uncertainty quantification, and targeted intervention design [6]. By explicitly comparing strengths, limitations, and application scenarios across modeling paradigms (Section “Comparative analysis of modeling approaches”), this study offers a structured decision-support perspective that is often missing from existing reviews. As a result, the manuscript not only summarizes the current state of infectious disease modeling but also proposes an application-oriented organizational framework that bridges theory, computation, and policy implementation.

The literature reviewed in this study was systematically collected from major scientific databases, including Web of Science, Scopus, PubMed, IEEE Xplore, and Google Scholar [7–11]. These platforms were selected to ensure comprehensive coverage of peer-reviewed journal articles, conference proceedings, and high-impact publications relevant to infectious disease modeling. A structured keyword-based search strategy was employed to identify relevant studies, ensuring broad coverage of deterministic, stochastic, network-based, and data-driven modeling approaches.

To ensure a transparent and systematic selection of relevant studies, a structured screening and inclusion process was adopted. Initially, all retrieved records were filtered based on title and abstract relevance to infectious disease modeling. Studies were included if they presented original mathematical, computational, or data-driven modeling frameworks, methodological innovations, or significant applications to epidemic dynamics. Review articles, tutorial papers, and highly cited benchmark studies were also retained to provide theoretical background and contextual coverage. Works were excluded if they lacked a clear modeling component, focused solely on clinical or biological experiments without mathematical analysis, or did not directly address epidemic dynamics. Additional filtering was performed to remove duplicate entries and low-impact or non-peer-reviewed sources. When multiple studies addressed similar modeling approaches, preference was given to more recent publications, widely cited contributions, and those demonstrating clear methodological rigor and relevance to public health applications. The structure of this paper is organized as follows. Section “Foundations of mathematical and computational approaches” outlines the foundations of mathematical and computational approaches. Section “Classical compartmental models” reviews classical compartmental models, while Section “Spatial and PDE-based epidemic models” discusses spatial and PDE-based epidemic models. Stochastic infectious disease models are presented in Section “Stochastic infectious disease models”. Section “Network-based epidemic models” examines network-based models and their applications, and Section “Data-driven and AI-based epidemic models” introduces data-driven modeling approaches. In section “Comparative analysis of modeling approaches” comparison of major infectious disease modeling approaches, highlighting their advantages, limitations, and typical application scenarios will be discussed. Finally, Section “Challenges, conclusion and future directions” provides concluding remarks, highlights key challenges, and outlines directions for future research.

## Foundations of mathematical and computational approaches

Mathematical modeling has long served as a cornerstone of infectious disease research, providing powerful tools for characterizing transmission dynamics and guiding public health interventions. A mathematical model offers an abstract yet systematic framework for representing epidemic processes through explicitly defined equations or rules, enabling rigorous investigation of disease spread and control. By expressing epidemiological mechanisms in mathematical form, researchers can identify key parameters, predict epidemic trajectories, and evaluate the potential impact of intervention strategies [12]. The reliability of such models depends on clearly stated assumptions, suitable structural choices, and validation against empirical data through analytical or numerical methods.

The foundations of this approach can be traced back to 1760, when Daniel Bernoulli introduced the first mathematical model to evaluate the effectiveness of smallpox variolation—an early milestone in linking quantitative analysis with public health decisions. Nearly a century later, in 1854, John Snow’s pioneering cholera mapping exemplified the power of data-driven epidemiology and spatial analysis in identifying disease sources.

Mathematical models are often expressed as systems of ordinary differential equations (ODEs) that describe the rates of change of key components, such as susceptible, infected, and recovered populations. Parameter estimation techniques are applied to calibrate these models, ensuring that theoretical predictions align with observed data [13, 14].

Classical deterministic models, such as the SIR, SIS, and SEIR formulations, originate from the foundational work of Kermack and McKendrick [15]. These compartmental models describe population-level disease dynamics using systems of ordinary differential equations, enabling the derivation of key epidemiological quantities such as the basic reproduction number ($R_0$). Over time, variants of these models have incorporated additional complexities, including demographic processes, nonlinear incidence rates, seasonal forcing, and disease-induced mortality [16, 17]. Despite their simplifying assumptions, deterministic models continue to provide valuable insights into equilibrium behavior, threshold conditions, and optimal control strategies.

Stochastic models extend this deterministic framework by accounting for the inherent randomness in infection and recovery processes. Such randomness becomes particularly significant in small populations or during the early stages of an epidemic, when chance events can determine whether an outbreak fades out or persists. Common stochastic formulations include continuous-time Markov chains, branching processes, and stochastic differential equations [18, 19]. These models generate probabilistic predictions and quantify uncertainty in epidemic outcomes, thereby complementing deterministic analyses.

Recognizing that real populations do not mix homogeneously, network and spatial models incorporate heterogeneity in contact patterns and mobility. Network-based frameworks represent individuals as nodes and interactions as edges, revealing how degree distributions, clustering, and network topology influence transmission thresholds and the effectiveness of interventions [4, 20]. Metapopulation and spatial models further capture geographic spread and regional coupling, providing insight into the effects of travel restrictions and localized containment strategies.

In recent years, hybrid and agent-based models (ABMs) have emerged as powerful computational approaches for simulating large-scale epidemics involving heterogeneous individuals and dynamic behaviors [21]. By integrating epidemiological mechanisms with behavioral, demographic, and mobility data, ABMs enable detailed evaluation of non-pharmaceutical interventions such as vaccination campaigns, contact tracing, and social distancing. Advances in computational methods—including efficient simulation algorithms, parallel computing, and data assimilation—have significantly enhanced the scalability and predictive capability of these models [22, 23].

Each modeling paradigm—deterministic, stochastic, network-based, and hybrid—offers distinct strengths. Deterministic models provide analytical tractability; stochastic models capture randomness and extinction probabilities; network models uncover structural determinants of transmission; and hybrid or agent-based approaches enable high-fidelity policy evaluation. The growing integration of mechanistic models with data-driven methods represents a promising direction for future research, fostering improved realism, uncertainty quantification, and decision support in epidemic forecasting and control.

### Study aim and objectives

The main aim of this review is to provide a comprehensive and critical assessment of mathematical and computational modeling approaches used to understand and control the spread of infectious diseases. It begins by tracing the historical development of epidemic models, outlining how early deterministic formulations laid the foundation for modern epidemiological analysis. The review examines the structure, assumptions, and limitations of classical compartmental frameworks such as the SIS and SIR models, emphasizing their continued relevance across diverse epidemic contexts. It also considers the distinction between deterministic and stochastic modeling approaches, highlighting their comparative strengths in representing predictable trends and inherent randomness in disease transmission.

Building on these foundations, the review synthesizes recent advancements in computational methodologies and data-driven modeling. In particular, it discusses how artificial intelligence and machine learning techniques have been integrated with mathematical models to improve parameter estimation, enhance forecasting accuracy, and support automated decision-making in epidemic management. The role of big data analytics and complex network theory is further explored, demonstrating how large-scale datasets and network-based representations of contact patterns can strengthen real-time surveillance, clarify transmission pathways, and guide rapid intervention strategies.

Finally, the review identifies key challenges and emerging opportunities in applying mathematical and AI-driven models to public health policymaking. Issues related to data quality, model uncertainty, interpretability, and interdisciplinary collaboration are underscored, alongside the growing importance of open-source datasets in promoting transparency, reproducibility, and wider accessibility of epidemic modeling tools. Collectively, these insights illustrate how integrated modeling approaches can inform evidence-based decision-making and support the development of effective, real-time epidemic response strategies.

Figure 1 presents a high-level overview of the main classes of infectious disease models examined in this survey, including compartmental, stochastic, spatial, network-based, and data-driven approaches, and highlights their conceptual interrelationships.

## Classical compartmental models

Deterministic models are widely used in epidemiology owing to their simplicity, interpretability, and computational efficiency. By employing fixed parameters to describe transitions between disease states, these models can predict infection dynamics at relatively low computational cost, making them accessible and practical tools for policymakers and public health authorities during outbreaks. They are particularly effective for studying large populations, where random fluctuations exert minimal influence on the overall epidemic trajectory [24].

Historically, deterministic epidemic models have played a pivotal role in understanding and controlling infectious diseases. For instance, during the 2014 Ebola outbreak, such models were crucial in elucidating epidemic progression and informing timely public health interventions. Similarly, studies of tuberculosis resurgence highlighted the importance of exogenous reinfection in sustaining disease persistence, influencing global control strategies. In the context of HIV/AIDS, deterministic frameworks evaluating universal testing and treatment have provided key insights that shaped international health policies.

Despite their analytical strength, deterministic models inherently neglect stochastic variability. This limitation becomes particularly significant in small populations or during the early stages of an outbreak, when random events can strongly affect transmission outcomes. To address these limitations, stochastic models are often employed alongside deterministic ones to incorporate randomness, yielding a more comprehensive and realistic description of epidemic dynamics.

Among deterministic formulations, the *compartmental modeling* framework remains the most prevalent. The classical SIR (Susceptible–Infected–Recovered) model partitions the population into compartments corresponding to distinct disease states, with transitions between them governed by systems of differential equations [17, 25]. This structure provides a systematic means of studying how infections emerge, spread, and subside, forming the theoretical foundation for a vast family of extended models now used in contemporary epidemiological research.

The conceptual origins of compartmental modeling trace back to the seminal work of Kermack and McKendrick (1927) [26], which established the mathematical basis of modern infectious disease theory. In general form, a compartmental model can be expressed as an autonomous dynamical system governed by ordinary differential equations (ODEs):

1 $$\dot{y}(t) = f(y(t)), \quad t > 0, \quad y(0) = y_0 \in \mathbb{R}^n,$$

where

$$\begin{aligned}&y = [y_1, y_2, \ldots, y_n]^\top : [0,\infty) \to \mathbb{R}^n, \cr& f = [f_1, f_2, \ldots, f_n]^\top : \mathbb{R}^n \to \mathbb{R}^n,\end{aligned}$$

and $\dot{y}$ denotes the time derivative of $y$. The function $f$ is assumed to satisfy the standard smoothness conditions that ensure existence and uniqueness of solutions to (1) (see, e.g. [27–29]).

Within this general framework, numerous epidemic models have been developed. Rather than explicitly modeling pathogen dynamics, these models track the temporal evolution of individuals across epidemiological compartments—such as susceptible, infected, and recovered populations. Canonical models including SI, SIS, and SIR systems provide a foundational yet powerful basis for exploring epidemic growth, evaluating intervention strategies, and characterizing the long-term behavior of infectious diseases.

### Susceptible–infected–susceptible Model

The *Susceptible–Infected–Susceptible (SIS)* model is one of the fundamental frameworks in mathematical epidemiology, used to describe infectious diseases in which recovery does not confer lasting immunity. Here, the susceptible population ($S$) represents individuals who are vulnerable to infection, while the infected population ($I$) represents individuals who are currently infectious. Upon recovery, individuals immediately return to the susceptible class, thereby remaining at risk of reinfection. Typical examples of diseases following this pattern include tuberculosis and gonorrhea, both of which have been effectively analyzed using SIS-type formulations [20, 30–33].

The same conceptual framework extends beyond biological epidemics to digital environments. In computer networks, for instance, it aptly captures the spread of computer viruses: an infected device can be “cured” by antivirus software, yet without continuous protection (e.g., an active scanner), it remains susceptible to reinfection by the same malware.

To incorporate contact heterogeneity, the $n$-*intertwined Markov model* was introduced as a network-based generalization of the classical SIS model [34]. This formulation allows the infection process to evolve on arbitrary, possibly directed, graphs and has proven particularly relevant for modeling complex systems such as human social networks and computer communication infrastructures.

Classical analyses of the this model typically assume *homogeneous mixing*, implying that every susceptible individual has an equal probability of encountering any infected individual [16, 35, 36]. However, real populations exhibit structured and limited contact patterns. Consequently, modern SIS formulations increasingly rely on *complex networks* that explicitly account for contact heterogeneity, clustering, and degree variability [37, 38].

The classical SIS model without demography (birth or death processes) is governed by the system of nonlinear ordinary differential equations:

2 $$\frac{dS}{dt} = -\frac{\beta S I}{N} + \gamma I, $$

3 $$\frac{dI}{dt} = \frac{\beta S I}{N} - \gamma I,$$

where $\beta$ denotes the transmission rate, $\gamma$ the recovery rate, and the total population $N = S + I$ is conserved. The infection term $\tfrac{\beta S I}{N}$ reflects the *mass-action principle*, while the recovery term $\gamma I$ describes the flow of individuals from the infected to the susceptible class.

**Epidemiological Threshold and Equilibria.** The basic reproduction number,

$$R_0 = \frac{\beta}{\gamma},$$

determines the qualitative behavior of the system:

- If $R_0 > 1$, the infection can invade and persist in the population, leading to a stable endemic equilibrium.
- If $R_0 \leq 1$, the disease-free equilibrium (DFE) is globally asymptotically stable, and the infection eventually dies out.

The equilibrium points of the this model are given by:

- **Disease-free equilibrium (DFE):**
  $$ (S^*, I^*) = (N, 0), $$
  representing the complete absence of infection.
- **Endemic equilibrium (for**$R_0 > 1$**):**
  $$ S^* = \frac{N}{R_0}, \qquad I^* = N \left(1 - \frac{1}{R_0}\right), $$
  corresponding to a nonzero steady state where the infection persists in the population.

**Fig. 2:**
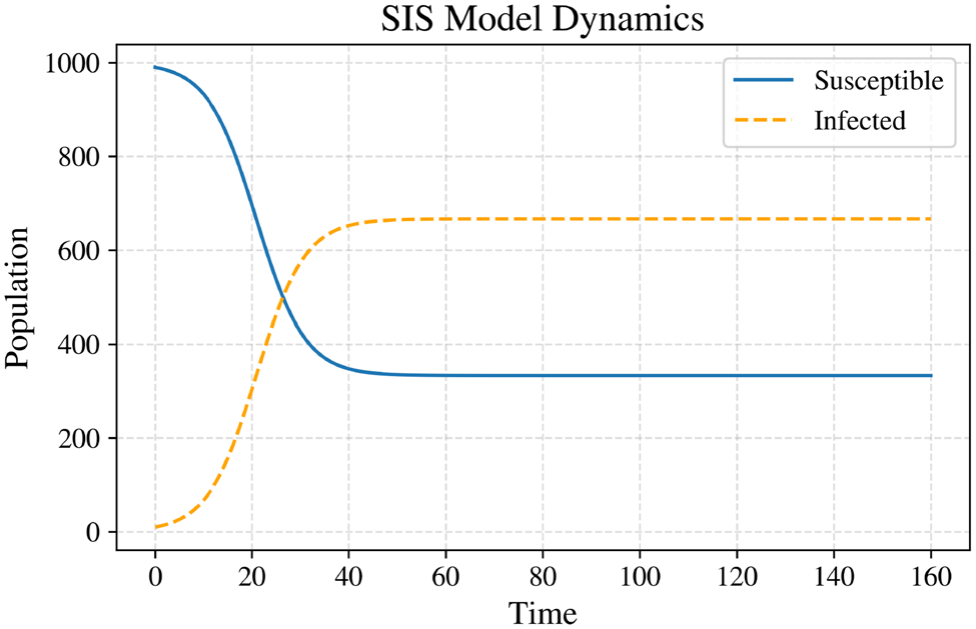
Graphical representation of the sis model showing transitions between susceptible ($S$) and infected ($I$) compartments. Parameters: infection rate $\beta = 0.3$, recovery rate $\gamma = 0.1$, and initial conditions $I(0) = 10$, $S(0) = N - I(0)$

This model therefore provides a simple yet powerful mathematical abstraction for systems characterized by repeated infections or reinfections, ranging from sexually transmitted diseases to computer malware propagation. The corresponding compartmental structure and transition mechanisms are schematically illustrated in Fig. 2.

### Susceptible–infected–recovered Model

The *Susceptible–Infected–Recovered (SIR)* model is a foundational deterministic framework introduced by Kermack and McKendrick (1927). It assumes that individuals who recover from infection acquire permanent immunity [15, 25]. Owing to its ability to capture the essential mechanisms of epidemic spread through a simple yet powerful mathematical formulation, it has been widely applied to the study of infectious diseases such as measles, influenza, and COVID-19.

It is expressed as a system of three nonlinear ordinary differential equations, the model provides a theoretical basis for understanding the temporal dynamics of disease transmission and for evaluating the potential impact of public health interventions. It also serves as a prototype for the development of more advanced epidemic models that incorporate additional compartments, population heterogeneity, or stochastic effects. In this framework, the total population is divided into three mutually exclusive compartments: the susceptible group ($S$), consisting of individuals who are at risk of contracting the disease; the infected group ($I$), representing individuals who are currently infectious and capable of transmitting the pathogen; and the recovered group ($R$), containing individuals who have recovered and acquired lasting immunity. The model operates under the following assumptions:

- The population is large and closed during the outbreak period; births, deaths, and migration are neglected.
- Infection is instantly transmissible, i.e., there is no latent or incubation phase.
- Recovered individuals acquire lifelong immunity and do not become susceptible again.
- The *mass-action principle* applies, implying homogeneous mixing in the population. The contact rate between susceptible and infected individuals is proportional to the product $SI$. Consequently, doubling either group doubles the number of new infections per unit time.

The dynamics of this model are governed by the following system of equations:

4 $$\frac{dS}{dt} = -\beta SI, $$

5 $$\frac{dI}{dt} = \beta SI - \gamma I, $$

6 $$\frac{dR}{dt} = \gamma I,$$

where $\beta > 0$ is the transmission rate and $\gamma > 0$ is the recovery rate. The mean infectious period is given by $D = 1/\gamma$. Since the total population $N = S + I + R$ remains constant, the model can be reduced to two equations.

**Fig. 3:**
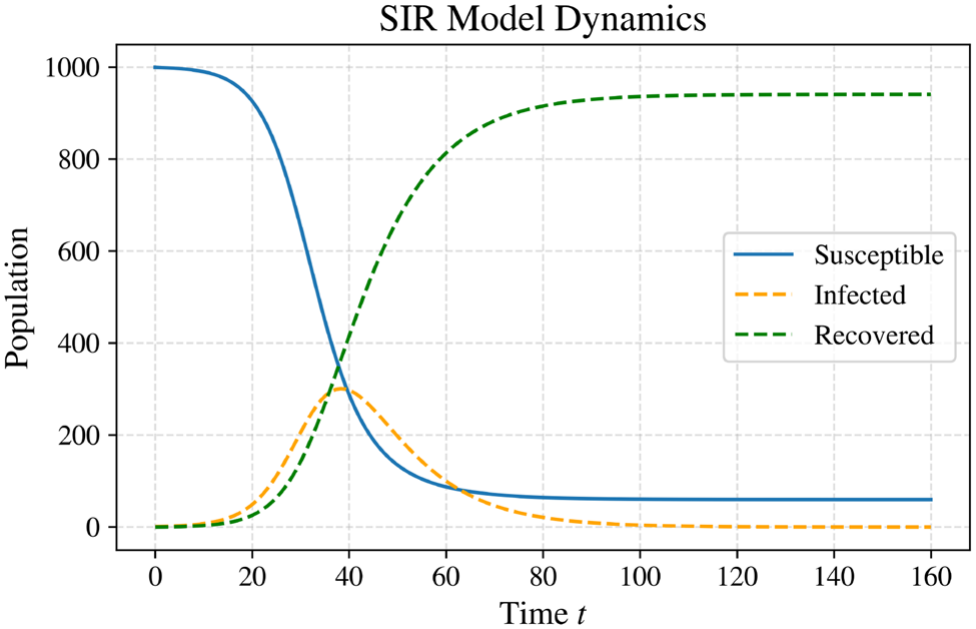
Graphical representation of the sir model showing transitions between susceptible ($S$), infected ($I$), and recovered ($R$) classes. Parameters: infection rate $\beta = 0.3$, recovery rate $\gamma = 0.1$, and initial conditions $I(0) = 1$, $R(0) = 0$, and $S(0) = N - I(0) - R(0)$

The bilinear term $\beta SI$ represents the rate of new infections per unit time under homogeneous mixing. The recovered population can be expressed as

$$R(t) = N - S(t) - I(t),$$

which allows simplification of the system. Figure 3 illustrates the compartmental structure of this model and the transitions between susceptible, infected, and recovered individuals.

In this model, susceptible individuals become infected at a rate proportional to the product of $S$ and $I$, while infected individuals recover at a rate $\gamma$ and transition to the recovered class. This framework is particularly suitable for diseases such as measles and influenza, where recovery provides long-term immunity.

The *effective reproduction number*, denoted by $R_e$, quantifies the average number of secondary infections caused by a single infected individual at time $t$, when only a portion of the population remains susceptible. It is defined as

$$R_e = \left( \frac{S(0)}{N} \right) \cdot \frac{\beta}{\gamma},$$

while the *basic reproduction number* ($R_0$) corresponds to the expected number of secondary infections produced by a single infected individual in a fully susceptible population, given by

$$R_0 = \frac{\beta}{\gamma}.$$

If the entire population is initially susceptible, with $S(0) = N - 1$, $I(0) = 1$, $R(0) = 0$, and $N$ sufficiently large, then

$$R_e = \left( \frac{N - 1}{N} \right) \cdot \frac{\beta}{\gamma} \approx R_0.$$

For large populations, the approximation $\tfrac{N - 1}{N} \approx 1$ holds. The behavior of the infected population $I(t)$ depends on the value of $R_e$:

- If $R_e \leq 1$, the number of infections decreases monotonically and eventually disappears as $t \to \infty$.
- If $R_e > 1$, the number of infections initially increases, reaches a peak, and then declines to zero as $t \to \infty$, representing an epidemic outbreak.

### The seir, sird, and SEIRS models

The simple yet fundamental SIR framework introduced in the previous section has provided valuable insights into the spread of infectious diseases within an idealized, randomly mixed susceptible population. To capture more realistic and complex disease transmission dynamics, the model can be extended by introducing additional compartments and transition pathways. These extended models—such as SEIR, SIRD, and SEIRS—allow for the inclusion of critical epidemiological factors such as latency (exposed individuals), recovery with mortality, loss of immunity, demographic changes, and age structure.

#### Seir Model (susceptible–exposed–infected–recovered)

The *Susceptible–Exposed–Infectious–Recovered (SEIR)* model extends the classical SIR framework by incorporating a biologically significant feature often neglected in simpler models—the latency or incubation period. In many infectious diseases, individuals who become infected do not immediately transmit the pathogen but pass through an *exposed* stage before becoming infectious. This modification makes the SEIR model more realistic for diseases such as COVID-19, SARS, measles, and chickenpox, where there is a delay between infection and infectiousness.

The model assumes homogeneous mixing, where all individuals are equally likely to interact. Transitions between compartments occur at constant rates, and the total population $N = S + E + I + R$ remains constant in the absence of births and deaths.

Numerous extensions of the SEIR model have been proposed to more accurately represent real-world complexities. For instance, nonlinear incidence rates have been introduced to capture behavioral responses and saturation effects in disease transmission. In [39], an SEIR model with nonlinear incidence was analyzed for COVID-19, establishing stability conditions for both the disease-free and endemic equilibria. Moreover, social and behavioral interventions have been integrated into SEIR frameworks; for instance [40], examined the effects of mask usage and reduced social gatherings, showing that these measures can significantly decrease transmission rates compared to complete lockdowns.

Other extensions include fractional-order SEIR models that incorporate memory effects and long-term dependencies [41], stochastic SEIR models that account for randomness in epidemic progression and extinction probabilities [42], and age-structured or network-based SEIR frameworks that capture heterogeneity in contact patterns [43]. Together, these developments have made SEIR-based modeling a cornerstone of modern epidemiological analysis.

The dynamics of the classical SEIR model are governed by the following system of nonlinear ordinary differential equations:

7 $$\frac{dS}{dt} = -\beta S I, $$

8 $$\frac{dE}{dt} = \beta S I - \sigma E, $$

9 $$\frac{dI}{dt} = \sigma E - \gamma I, $$

10 $$\frac{dR}{dt} = \gamma I,$$

where $\beta$ is the transmission rate, $\sigma$ is the rate at which exposed individuals become infectious (the inverse of the incubation period), and $\gamma$ is the recovery rate. The mean latent period is $1/\sigma$, and the mean infectious period is $1/\gamma$.

#### Sird Model (susceptible–infected–recovered–deceased)

The SIRD model extends the SIR framework by introducing a *deceased* compartment to account for disease-induced mortality. It categorizes the population into four groups: susceptible ($S$), infected ($I$), recovered ($R$), and deceased ($D$). This framework is especially relevant for diseases in which mortality significantly influences transmission dynamics and informs public health planning.

This framework is especially relevant for diseases in which mortality has a substantial influence on transmission dynamics and public health planning. The model, derived from the foundational work of Kermack and McKendrick (1927) [15], has been used for high-fatality diseases such as Ebola and COVID-19 [17, 44]. For instance, Anastassopoulou et al. [45] applied the SIRD model to COVID-19 data to estimate key parameters, while [46] incorporated time-dependent transmission rates to capture lockdown effects.

The classical SIRD model is described by:

11 $$\frac{dS}{dt} = -\beta S I, $$

12 $$\frac{dI}{dt} = \beta S I - \gamma I - \mu I, $$

13 $$\frac{dR}{dt} = \gamma I, $$

14 $$\frac{dD}{dt} = \mu I,$$

where $\beta$ is the transmission rate, $\gamma$ is the recovery rate, and $\mu$ is the mortality rate. The total population at any time is $N = S + I + R + D$.

#### SEIRS Model (susceptible–exposed–infected–recovered–susceptible)

The SEIRS model extends the SEIR framework by allowing recovered individuals to lose immunity and return to the susceptible class, making it suitable for diseases where immunity is temporary, such as influenza, pertussis, and certain coronaviruses.

Hethcote [16] provided the mathematical foundation for this model, which introduces a waning immunity rate $\omega$. The model equations are:

15 $$\frac{dS}{dt} = -\beta S I + \omega R, $$

16 $$\frac{dE}{dt} = \beta S I - \sigma E, $$

17 $$\frac{dI}{dt} = \sigma E - \gamma I, $$

18 $$\frac{dR}{dt} = \gamma I - \omega R,$$

where $\omega$ represents the rate at which recovered individuals lose immunity and re-enter the susceptible pool.

## Beyond the classical and refinements of incidence and contact functions

A central aspect of epidemic modeling is the representation of *new infections*. In the classical SIR framework, the incidence term is bilinear, given by $\beta SI$, implying that infections increase linearly with both susceptible and infectious individuals. This assumption is most accurate in the early stages of an outbreak, when prevalence is low and behavioral responses are minimal. In practice, however, transmission may *saturate* due to limits on contact opportunities, or decrease as individuals adopt protective behaviors. To address these shortcomings, models often replace $\beta SI$ with nonlinear terms of the form $g(I)S$, where $g(I)$ encodes saturation, crowding, or psychological effects. Such modifications lead to *saturated incidence* and *standard incidence* formulations [47–58], which better capture epidemic plateaus, nonlinear growth, and the eventual decline in infection rates.

Similarly, the modeling of contact rates has been refined. Instead of assuming proportionality with the total population size $N$, more realistic formulations introduce saturating functions $C(N)$, fractional powers of $N$, or mechanistic contact structures such as pair formation [59–63]. These generalizations incorporate limits to social interactions and network saturation, and they help explain long-term dynamics such as recurrent outbreaks or endemic equilibria.

### Structured population models

Real populations are not homogeneous, but vary across age, geography, and social networks. Incorporating such heterogeneity is critical for producing models that are both realistic and policy-relevant. Several structured extensions of compartmental models have been widely adopted:

- **Age-structured models** account for age-dependent susceptibility, infectivity, and recovery rates, as well as the impact of age-specific interventions such as school closures or vaccination campaigns [64, 65]. These models are particularly valuable for diseases like influenza and COVID-19, where contact patterns are strongly age-dependent.
- **Spatially structured models** incorporate geographic heterogeneity and mobility. By embedding compartments into networks or partial differential equations, these models capture local outbreaks, wave-like spatial spread, and the role of travel restrictions in slowing epidemics [65, 66].
- **Models with additional compartments** extend the classical SIR structure to include more epidemiological detail. Common examples include SEIR models (with an exposed but not yet infectious class), models with waning immunity or reinfection, and frameworks incorporating interventions such as vaccination, isolation, or quarantine [67, 68]. These additions allow models to represent realistic immunological and behavioral processes, enabling more precise evaluation of public health strategies.

By capturing population structure, these models enable analysis of targeted interventions, such as prioritizing vaccine allocation or optimizing mobility restrictions, which would be overlooked in unstructured frameworks.

### Advanced mathematical frameworks

Beyond refining incidence and adding structural detail, epidemic models have been generalized to more advanced mathematical formulations that reflect temporal complexity, memory, and randomness:

- *Delayed models* incorporate time lags arising from incubation periods, temporary immunity, or behavioral feedbacks. Delay differential equations allow epidemics to exhibit oscillatory dynamics, stability switches, and richer transient behavior. These models are highly relevant for infections such as influenza or HIV, where delays play a decisive role [69–80].
- *Fractional-order models* replace integer-order derivatives with fractional operators, providing a natural framework to capture memory and hereditary effects. They can reproduce long-term persistence, anomalous diffusion, and sub-exponential epidemic growth patterns observed in COVID-19 and other diseases [69, 81–85]. Such models link epidemiology with complex systems theory and have become an active research frontier.
- *Stochastic models* extend deterministic ODEs by incorporating demographic randomness, fluctuating contact processes, or environmental noise. They are indispensable for studying disease emergence in small populations, extinction probabilities, or rare but impactful events such as superspreading [86–93]. Stochastic frameworks also bridge epidemic modeling with probability theory, enabling rigorous analysis of risk and uncertainty.

Together, these advanced frameworks enrich epidemic modeling, enabling researchers to explore not only average outcomes but also the variability and uncertainty inherent in real epidemics.

From nonlinear incidence laws and structured populations to delayed, fractional, and stochastic dynamics, modern epidemic modeling demonstrates how the classical SI/SIR framework can evolve into a versatile set of mathematical tools. These refinements not only deepen theoretical understanding of epidemic processes but also provide actionable insights for policymakers. By combining mechanistic detail with statistical realism, extended epidemic models are increasingly central to forecasting, intervention design, and global health preparedness.

### Applications of epidemic and compartmental models

Over the past decades, epidemic and compartmental models have transcended their original role of describing infectious disease outbreaks. Their structured, quantitative representation of populations, interactions, and interventions has enabled applications in diverse domains, from virology and chronic diseases to ecology, veterinary medicine, and even the modeling of digital and social contagions. The versatility of these frameworks lies in their ability to capture dynamic processes in populations, evaluate intervention strategies, and provide theoretical insights that inform public health and policy decisions.

#### Applications in infectious diseases

The earliest and most influential applications of compartmental models involve *infectious diseases*. Classical virus dynamics and epidemic spread models [17, 72, 85, 94–98] introduced fundamental epidemiological concepts such as the epidemic threshold and the basic reproduction number $R_0$, which remain central for assessing the effectiveness of interventions like vaccination, treatment, and quarantine.

For *influenza* [99–103], models have captured both seasonal and pandemic dynamics, incorporating waning immunity, vaccination programs, and antigenic drift. They have guided the design of interventions such as vaccination schedules, social distancing, travel restrictions, and the stockpiling of antivirals. The *SARS epidemic (2002–2003)* [104–109] highlighted the role of super-spreading events, showing that rapid case isolation, quarantine, and contact tracing could contain outbreaks even in the absence of pharmaceutical countermeasures.

Models of *Ebola virus disease* [110–115] emphasized hospital-based transmission, unsafe burial practices, and community contact patterns. They demonstrated that targeted interventions such as safe burial protocols, improved case isolation, and healthcare worker protection significantly reduce transmission.

For chronic infectious diseases such as *hepatitis B* and *C* [116–128] and *tuberculosis* [129–138], modeling efforts focus on long-term persistence, latency, reinfection, and drug resistance. These models inform vaccination campaigns, preventive therapy strategies, and responses to multi-drug resistant strains.

Vector-borne infections represent another important application area. Models of *vector-borne diseases* [139–145] such as *malaria* [146–156], *dengue* [157–163], and *Zika virus* [161, 164–173] explicitly represent host–vector interactions, immunity dynamics, climate variability, and spatial heterogeneity. These studies have been instrumental in shaping strategies such as insecticide-treated bed nets, mosquito control programs, and vaccine deployment.

Highly transmissible diseases like *measles* [174] and *COVID-19* [155, 175–207] illustrate the importance of combining traditional compartmental models with agent-based and network-based approaches. These models capture heterogeneity in contact patterns, vaccination strategies, and non-pharmaceutical interventions, supporting decision-making at both local and global levels.

Finally, *HIV/AIDS* models [208–215] have been instrumental in understanding chronic infection dynamics, assessing the long-term effects of antiretroviral therapy, and guiding prevention policies including condom use, pre-exposure prophylaxis, and public health campaigns.

### Applications beyond infectious diseases

The influence of epidemic modeling extends far beyond classical infectious disease contexts. For example, in chronic non-communicable diseases, models of *diabetes mellitus* [134, 216] describe glucose–insulin regulation, treatment adherence, and long-term complications. Similarly, *cancer and tumor invasion* models [217] apply compartmental approaches to describe tumor growth, angiogenesis, immune interactions, and responses to therapy. Models of *human papillomavirus (HPV)* and its link to *cervical cancer* [218] inform vaccination and screening strategies.

In veterinary and agricultural domains, *animal disease models* [85, 219–221] have been vital for controlling epidemics such as foot-and-mouth disease, bovine tuberculosis, and avian influenza, where economic and food-security consequences are severe.

In microbiology and ecology, *chemostat models* [222–225] describe microbial growth, nutrient dynamics, and competition, with applications ranging from environmental ecology to industrial bioprocess engineering. These models illustrate how the mathematics of epidemic spread also applies to microbial communities.

The conceptual reach of epidemic modeling extends even into *digital and social systems*. Models of *computer viruses and rumor spreading* [49, 226–238] draw analogies between pathogen transmission and information diffusion, providing insights into cybersecurity, misinformation control, and viral marketing.

Likewise, epidemic frameworks have been adapted to study *addictions and social epidemics*, where behaviors spread through peer influence and social reinforcement. Models of *alcohol use* [239–249], *tobacco consumption* [250–255], *opioid and heroin addiction* [252, 256–263], *cocaine use* [264, 265], and broader issues such as *drug epidemics and obesity* [266, 267–273] model relapse dynamics, peer influence, and intervention strategies, treating these behaviors as “social contagions.”

Epidemic and compartmental modeling has thus emerged as a unifying framework capable of representing dynamic processes across health, ecological, social, and digital domains. By abstracting the essential mechanisms of transmission, interaction, and intervention, these models not only deepen theoretical understanding but also provide practical tools for designing strategies, guiding policies, and mitigating risks in diverse application areas.

## Spatial and PDE-based epidemic models

Spatial epidemic models explicitly incorporate the geographic distribution of populations and the heterogeneity of disease transmission across space. Unlike classical compartmental frameworks, which assume homogeneous mixing, spatial models recognize that individuals interact locally and may move or diffuse across regions. This spatial structure is particularly important for capturing realistic outbreak patterns, localized clustering, and the spread of infection fronts.

A common mathematical approach is to employ *reaction–diffusion partial differential equations (PDEs)*. In these systems, the *reaction terms* represent local processes such as infection, recovery, birth, and death, while the *diffusion terms* describe the movement or dispersal of individuals across space. Reaction–diffusion epidemic models have successfully reproduced phenomena such as traveling waves of infection, patchy persistence, and recurrent localized outbreaks [3, 274–276].

### General reaction–diffusion epidemic Model

Let $\Omega \subset \mathbb{R}^m$ denote a bounded spatial domain with smooth boundary $\partial \Omega$. A general reaction–diffusion epidemic model is given by

19 $$\frac{\partial u}{\partial t}(x,t) = D \Delta u(x,t) + f(u(x,t),x), \quad x \in \Omega, \; t > 0,$$

with initial condition

20 $$u(x,0) = u_0(x), \quad x \in \Omega,$$

and homogeneous Neumann (no-flux) boundary condition

21 $$\frac{\partial u}{\partial \nu}(x,t) = 0, \quad x \in \partial \Omega, \; t > 0.$$

Here, $u(x,t) = (u_1(x,t), \ldots, u_n(x,t))^\top$ represents compartment densities (e.g., $S$, $I$, $R$), $D = \mathrm{diag}(d_1,\ldots,d_n)$ is the diffusion matrix with mobility rates $d_i$, $f(u,x)$ encodes nonlinear local dynamics, and $\nu$ is the outward unit normal on $\partial \Omega$.

This framework generalizes classical ODE epidemic models to spatially structured populations, enabling analysis of persistence, invasion speeds, and the effect of heterogeneous environments.

**Examples of reaction–diffusion epidemic models**

Allen [19] proposed an SIS model in heterogeneous environments:

22 $$\frac{\partial S}{\partial t}(t,x) = d_S \Delta S - \beta(x)\,\frac{S(t,x)I(t,x)}{S(t,x)+I(t,x)} + \gamma(x) I(t,x), $$

23 $$\frac{\partial I}{\partial t}(t,x) = d_I \Delta I + \beta(x)\,\frac{S(t,x)I(t,x)}{S(t,x)+I(t,x)} - \gamma(x) I(t,x),$$

with Neumann boundary conditions ensuring no flux across the domain boundary.

Similarly, a spatially explicit SIR system can be written as

24 $$\frac{\partial S}{\partial t}(t,x) = d_S \Delta S + b(x) - \beta(x) S(t,x) I(t,x) - \mu(x) S(t,x), $$

25 $$\frac{\partial I}{\partial t}(t,x) = d_I \Delta I + \beta(x) S(t,x) I(t,x) - (\mu(x)+\gamma(x)) I(t,x), $$

26 $$\frac{\partial R}{\partial t}(t,x) = d_R \Delta R + \gamma(x) I(t,x) - \mu(x) R(t,x),$$

where $$d_S, d_I, d_R$$ are diffusion coefficients and $$b(x), $$ $$\beta(x), $$ $$\mu(x), \gamma(x)$$ encode spatially dependent vital and epidemiological rates.

We implemented a Python solver for SIS and SIR reaction–diffusion PDEs with spatial heterogeneity. The solver integrates the dynamics using a forward Euler scheme with Neumann boundary conditions, implemented via mirrored ghost nodes to enforce zero flux at the boundaries. Default parameters include:

- Diffusion rates $$d_S = d_I = d_R = 0.02$$,
- Infection rate $\beta_0 = 0.6$, recovery rate $\gamma_0 = 0.2$,
- Mortality $\mu_0 = 0.0$ and birth rate $b_0 = 0.0$ (SIR only).

**Fig. 4:**
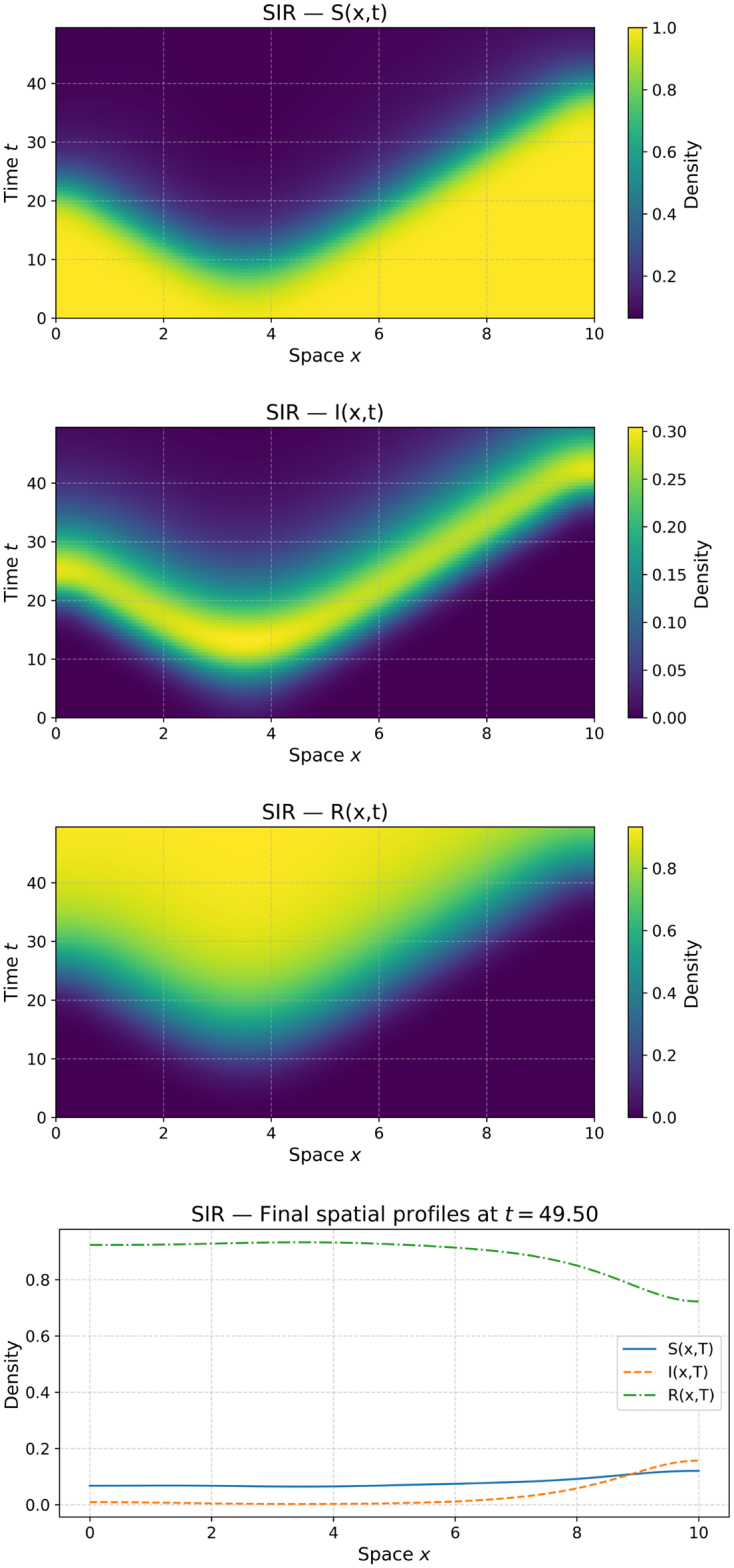
Simulation of SIS/SIR reaction–diffusion epidemic models with Neumann boundary conditions. The solver produces spatio-temporal heatmaps for $S,I,R$ compartments and the final spatial profiles

The spatial domain $[0,L]$, with $L=10.0$, is discretized using $N=201$ grid points, yielding a spatial resolution of $\Delta x \approx 0.05$. The time step is chosen to satisfy the CFL stability condition, $\Delta t \leq 0.45 \Delta x^2 / \max(d_S,d_I,d_R)$. Simulations are performed up to a final time of $T=50.0$ and generate heatmaps of compartment densities as well as final spatial profiles (see Fig. 4).

### Applications and extensions of pde models

Reaction–diffusion epidemic models have been applied to a wide range of real-world scenarios, including influenza, vector-borne infections such as malaria and dengue, and COVID-19. They are particularly well-suited for analyzing *spatially targeted interventions*, such as localized vaccination, travel restrictions, quarantine zones, and adaptive mobility policies [277, 278].

Extensions of these PDE frameworks include:

- *Metapopulation PDE models*, where the domain is divided into subregions connected by diffusion or migration [279, 280].
- *Heterogeneous diffusion rates*, allowing different compartments or subgroups to have distinct mobility patterns (e.g., humans vs. vectors).
- *Hybrid PDE–network models*, coupling continuous spatial spread with discrete social or travel networks.
- *Nonlocal diffusion*, incorporating commuting kernels or long-range travel to capture modern mobility patterns [281].
- *Fractional-order models*, e.g. in [282] a classical and fractional-order mathematical model for Buruli ulcer transmission dynamics using real epidemiological data from Cameroon, demonstrating the effectiveness of hybrid modeling frameworks that combine parameter estimation, sensitivity analysis, and fractional calculus has been developed.

Recent advances include studies on persistence, eradication strategies, and stability of equilibria. For example, Peng [283] analyzed the impact of heterogeneous diffusion on SIS persistence, while Peng and Yi [284] extended the model to multi-risk domains. Huang et al. [285] investigated SIS systems under Dirichlet boundaries, proving global stability results. Liu and Cui [286] examined SIRS models with saturated incidence, and Chang et al. [287] introduced sparse optimal control into SIR PDEs. Lou and Salako [288] studied spreading speeds and equilibria, while Mammeri [289] developed a diffusive SEIR model for COVID-19 dynamics in France.

Applications extend further: Lin et al. [290] proposed a nonlocal PDE for West Nile virus, Zhang et al. [291] developed an SVIR model in heterogeneous domains, Cherniha and Davydovych [292] formulated age-structured diffusive PDEs, and Wang et al. [293] introduced a nonlinear SEIR system with explicit super-spreader compartments.

Spatial and PDE-based epidemic models provide a rigorous framework for understanding how infections spread across geographic landscapes. By combining local nonlinear reactions with spatial diffusion or nonlocal movement, these models explain traveling waves, patchy persistence, and heterogeneous outbreak patterns. Their ability to incorporate targeted interventions, complex mobility, and hybrid structures makes them indispensable for modern epidemiology, bridging the gap between classical compartmental ODE models and real-world epidemic dynamics.

#### COVID-19 models

Spatially explicit and PDE-based approaches have been widely employed to study the spread of COVID-19, incorporating relapse, immunity, vaccination, and logistics into epidemic dynamics.

In [294], a delayed COVID-19 model in heterogeneous environments was proposed, incorporating relapse and quarantine. Using the global exponential attractor method, the authors demonstrated that the leading eigenvalue $\lambda^*$ provides a more accurate threshold indicator for disease persistence than the classical basic reproduction number $R_0$. Similarly [295], introduced a reaction–diffusion framework with humoral immunity and proved that spatial viral diffusion plays a critical role in determining long-term persistence of infection. Extending this idea [296], studied a model with *nonlocal dispersal* and vaccination, deriving the basic reproduction number $R_0$ via the next-generation operator and showing how dispersal patterns influence control strategies. A continuum-mechanics reformulation of epidemic PDEs was provided in [297], offering simulations in both one- and two-dimensional domains that highlight the importance of geometric and spatial structure in epidemic dynamics.

More recently, several works have integrated vaccination policies directly into PDE epidemic models. For example [298], developed a multi-compartment *reaction–diffusion SEIR-type PDE* with vaccination and patient isolation represented as distributed control variables. Solving a multi-objective optimal control problem, they showed that spatially heterogeneous vaccination strategies are significantly more effective than uniform ones, particularly near advancing epidemic wavefronts. In a complementary study [299], modeled the *transport and distribution dynamics of vaccine supply* using a diffusion-type PDE coupled with an epidemic system, demonstrating how delivery constraints and spatial fluxes reshape feasible allocation policies. At a more microscopic scale [300], employed a *kinetic PDE approach* based on Boltzmann-type equations to incorporate social-structural heterogeneity in contact behavior and compliance. Their results underscored how differences in individual risk perception and adherence to interventions affect the marginal benefits of targeted vaccination. Recent advances in infectious disease modeling have further motivated the present study. The authors in [301] proposed a fractional-order SEAIHR compartmental model to analyze the transmission dynamics of COVID-19 in Cameroon while explicitly accounting for enhanced immune responses and booster immunity. Their results demonstrated that fractional-order models can provide improved accuracy in capturing epidemic dynamics and parameter sensitivity.

Together, these studies illustrate the central role of PDE-based models in analyzing COVID-19. By capturing spatial spread, intervention heterogeneity, and logistical constraints, PDE frameworks provide unique insights into both epidemic control and vaccine distribution strategies during large-scale outbreaks.

#### Vector-borne and environmentally mediated diseases

For malaria [277], analyzed heterogeneous transmission incorporating vector bias and seasonal fluctuations, deriving threshold conditions in terms of $R_v$ and $R_0$. For Zika virus [302], proposed a degenerate reaction–diffusion framework with environmental mosquito transmission, proving global attractivity of the disease-free state using Lyapunov functions. These studies illustrate how diffusion and environmental drivers critically shape vector-borne epidemics.

#### Hepatitis B virus (HBV)

Spatial PDE models have also been developed for chronic viral infections such as HBV. In [303], diffusion of free virions was introduced, and the existence of traveling wave solutions was established. [304] incorporated logistic hepatocyte proliferation into a PDE framework, proving well-posedness and global stability. The role of non-cytopathic antiviral mechanisms was highlighted in [305], where their inclusion promoted viral clearance. Finally [306], examined SIRS systems with nonlinear incidence and partial immunity, showing that spatial heterogeneity increases epidemic risk but diffusion can mitigate persistence.

#### Generalized SEIRS and SIS systems

Several works extend classical SEIRS and SIS dynamics to spatial settings. [307] analyzed SEIRS models with heterogeneous recovery, demonstrating that exposed individuals’ movement strongly influences persistence. [308] proposed cross-diffusion SI systems, proving the existence of coexistence states and Turing-type instabilities that generate spatial pattern formation.

#### Nonlinearities, advection, and free-boundary dynamics

More general frameworks introduce nonlinear effects, advection, and moving boundaries. [286] investigated SIRS models with saturated incidence, showing that strong saturation can suppress outbreaks. In contrast [309], studied SIR models with Holling type II treatment, proving that epidemics may persist even when $R_0 < 1$. Avian–human influenza PDEs were analyzed in [310], yielding global stability results, while the MEM framework of [311] coupled PDEs and ODEs with sanitation feedback. [312] showed that advection processes can spatially concentrate infections downstream, altering persistence conditions. West Nile virus dynamics were modeled in [313] with nonlocal and time-delayed PDEs, where explicit epidemic thresholds were derived. Reaction–advection–diffusion models with free boundaries were introduced in [314], defining a spatio-temporal risk index $R_0^F(t)$ that governs disease invasion. Finally [315], reviewed how landscape heterogeneity and host mobility, using rabies as a case study, modify epidemic spread in fragmented habitats.

### Synthesis

Collectively, these studies highlight three overarching themes:

1. *Threshold dynamics are reshaped by spatial processes* such as diffusion, advection, and nonlocal dispersal, leading to new persistence and extinction criteria.
2. *Spatial heterogeneity drives clustering and asymmetry*, producing phenomena such as downstream concentration, patchiness, or wavefront formation.
3. *Control measures interact nonlinearly with space*, where saturation effects, antiviral mechanisms, or cross-species transmission alter intervention success beyond the predictions of classical $R_0$.

In summary, reaction–diffusion, advection, and nonlocal epidemic models extend classical epidemiology by embedding geography, mobility, and heterogeneity into the dynamics. They provide rigorous tools for designing spatially targeted interventions and improving preparedness for emerging infectious diseases.

### Translating epidemic models into public health policy

Recent public health emergencies provide concrete evidence that mathematical models have directly influenced policy decisions. During the COVID-19 pandemic, transmission models developed by the Imperial College COVID-19 Response Team (e.g., CovidSim) were used to project healthcare demand and mortality under alternative non-pharmaceutical intervention scenarios. These projections informed the timing and intensity of national lockdown policies in several countries by quantifying potential hospital overload and mortality reductions under mitigation and suppression strategies [316]. Similarly, in the United States, scenario-based ensemble models coordinated through the CDC COVID-19 Scenario Modeling Hub were incorporated into advisory discussions on booster vaccination strategies and healthcare capacity planning. Importantly, policymakers were presented with uncertainty ranges and multiple plausible epidemic trajectories rather than single-point forecasts, enabling risk-aware decision-making under incomplete data [317].

Beyond pandemic response, real-world implementation of epidemic modeling has also shaped vaccination prioritization and resource allocation strategies. In Colombia, national health authorities collaborated with academic modeling groups to develop real-time forecasting and cost-effectiveness analyses that guided vaccine rollout sequencing and regional allocation strategies [318]. In New Zealand, stochastic epidemic simulations produced and has been used to evaluate elimination versus mitigation strategies and supported early border control and suppression policies. Government officials explicitly acknowledged that model outputs were interpreted alongside socioeconomic constraints and healthcare system capacity, highlighting the role of modeling as a decision-support tool rather than a prescriptive mechanism [319]. These examples underscore the practical utility of mathematical models while also illustrating how uncertainty, data limitations, and operational constraints shape their interpretation in applied policy contexts.

## Stochastic infectious disease models

Deterministic epidemic models describe a single, noise-free trajectory of disease spread under fixed transition rates. While these models provide an important baseline for understanding average epidemic behavior, they fail to capture the intrinsic randomness that governs real-world transmission processes. *Stochastic* epidemic models explicitly incorporate random fluctuations and are therefore particularly valuable in three epidemiologically relevant contexts: (i) small or closed populations such as households, schools, or island communities; (ii) the *early phase* of an outbreak, when the number of infectious individuals is low and chance events can determine outbreak success or extinction; and (iii) the analysis of outbreak-size distributions, extinction probabilities, and long-term endemic variability [16, 17, 44, 320, 321].

From a public health perspective, these capabilities enable stochastic models to quantify uncertainty, estimate the probability of epidemic emergence, and evaluate the robustness of intervention strategies under realistic variability. Such information is critical for risk-based decision-making, early warning systems, and contingency planning, particularly during emerging outbreaks where data remain sparse and noisy.

Two principal sources of randomness are typically represented:

- *Gaussian fluctuations*, representing small, continuous perturbations arising from demographic variability or reporting noise, commonly modeled as white noise in Itô stochastic differential equations (SDEs);
- *Jump processes*, capturing rare but high-impact events such as super-spreading incidents, mass gatherings, sudden behavioral shifts, or abrupt mobility changes, typically represented by Lévy noise.

Across the SIS, SIR, SIRS, and SEIR model families—and in intervention-augmented variants such as SIQR or SIQS—stochastic formulations refine epidemic thresholds, elucidate noise-induced extinction near criticality, and provide rigorous long-run characterizations such as ergodicity and persistence in the mean [42, 322–326]. These properties support more realistic projections of epidemic uncertainty bands, enabling policymakers to evaluate best-case and worst-case intervention scenarios rather than relying solely on mean-field predictions.

### Core modeling frameworks

#### Discrete-time markov chains (DTMCs)

In a DTMC framework, time evolves in discrete increments and the states represent integer counts of individuals in each compartment. This formulation aligns naturally with routinely reported surveillance data, which are typically aggregated on daily or weekly time scales.

For an SIS process with $I(t) = i$ infectives in a population of size $N$, the transition probabilities over a small time step $\Delta t$ are

$$p_{ji}(\Delta t) =\begin{cases}\dfrac{\beta i(N-i)}{N}\,\Delta t, & j = i+1, \\(b+\gamma)i\,\Delta t, & j = i-1, \\1-\Big(\tfrac{\beta i(N-i)}{N}+(b+\gamma)i\Big)\Delta t, & j = i, \\0, & \mathrm{otherwise}.\end{cases}$$

Here $b(i)=\tfrac{\beta}{N}i(N-i)$ denotes the infection rate and $d(i)=(b+\gamma)i$ the recovery rate. The disease-free state is absorbing, implying eventual extinction with probability one [320].

DTMC models are especially useful for short-term outbreak forecasting and probabilistic risk assessment in settings where data are reported at discrete intervals, such as hospital admission records or case notification systems.

#### Continuous-time markov chains (CTMCs)

In CTMC models, time is continuous while state variables remain integer-valued, allowing a more faithful representation of event-driven transmission dynamics. For the SIS process, the transition probabilities over $\Delta t$ satisfy

$$\begin{aligned}&p_{ji}(\Delta t) \cr&=\begin{cases}b(i)\,\Delta t + o(\Delta t), & j = i+1, \\d(i)\,\Delta t + o(\Delta t), & j = i-1, \\1 - [b(i) + d(i)]\,\Delta t + o(\Delta t), & j = i, \\o(\Delta t), & \mathrm{otherwise},\end{cases}\end{aligned}$$

leading to the Kolmogorov forward equations

$$\frac{dp_i}{dt} = p_{i-1}b(i-1) + p_{i+1}d(i+1) - p_i[b(i)+d(i)].$$

CTMCs form the theoretical basis of exact stochastic simulation techniques such as the *Gillespie algorithm* [16, 17], which are widely used for scenario testing, intervention benchmarking, and synthetic outbreak generation for preparedness exercises.

#### Stochastic differential equations (SDEs)

For large populations ($N \gg 1$), diffusion approximations yield Itô-type SDEs that provide computationally efficient continuous approximations of discrete stochastic processes. For the SIS model:

$$\begin{aligned}dI(t) &= \Big(\tfrac{\beta}{N}I(N-I) - (b+\gamma)I\Big)\,dt\cr&\quad+ \sqrt{\tfrac{\beta}{N}I(N-I)+(b+\gamma)I}\, dW_t,\end{aligned}$$

where $W_t$ denotes a Wiener process.

For the SIR system with $X(t) = (S,I)^\top$, one obtains

27 $$dS = -\tfrac{\beta}{N}SI\,dt + B_{11}\,dW_1 + B_{12}\,dW_2, $$

28 $$dI = \Big(\tfrac{\beta}{N}SI - \gamma I\Big)\,dt + B_{21}\,dW_1 + B_{22}\,dW_2.$$

From an applied perspective, SDE models enable uncertainty quantification and ensemble forecasting, which are essential for public health planning under incomplete or noisy surveillance data. They also allow data assimilation techniques, such as particle filtering and Bayesian inference, to update epidemic forecasts in near real time.

#### Jump–diffusion models

To capture abrupt, high-impact shocks—such as super-spreading events, natural disasters, mass gatherings, or sudden policy changes—SDEs are extended by adding compensated Poisson jump terms:

$$\begin{aligned}dX(t) &= f(X(t))\,dt + G(X(t))\,dW_t \cr&\quad+ \int_{\mathbb{R}} H(X(t^-),z)\,\tilde N(dt,dz),\end{aligned}$$

where $\tilde N$ is a compensated Poisson random measure.

These jump–diffusion formulations introduce discontinuities that can significantly alter epidemic trajectories and modify persistence and extinction thresholds [327–330]. In practical terms, they provide a quantitative framework for stress-testing intervention strategies against worst-case outbreak scenarios.

#### Branching process approximations

During the early phase of an epidemic ($I \ll N$), transmission dynamics are well approximated by a Galton–Watson branching process. The extinction probability $q$ satisfies

$$q = G(q),$$

where $G$ denotes the offspring generating function. This formulation establishes a direct probabilistic connection between outbreak risk and the basic reproduction number $R_0$ [17, 331]. Such approximations are widely used to estimate the probability that imported cases will seed sustained community transmission.

#### Agent-based and Monte Carlo Models

Beyond Markovian assumptions, agent-based models (ABMs) and Monte Carlo simulations capture heterogeneity in contact networks, individual mobility, compliance behavior, and demographic structure. These models have been extensively applied to evaluate non-pharmaceutical interventions, vaccination prioritization, and contact tracing strategies in complex, realistic settings [1, 332]. Although computationally intensive, ABMs provide high-resolution policy insight when detailed mobility or social contact data are available.

In ABM frameworks, agents follow predefined behavioral rules governing movement, contact formation, infection, recovery, and response to interventions. This bottom-up modeling approach allows complex macroscopic epidemic patterns to arise from simple individual-level interactions. Consequently, ABMs are particularly effective in capturing nonlinear transmission dynamics, superspreading events, spatial mobility, and feedback mechanisms between disease spread and human behavior.

A major advantage of ABMs lies in their ability to evaluate non-pharmaceutical interventions and targeted control strategies. Policies such as social distancing, quarantine, school closures, contact tracing, and vaccination prioritization can be explicitly implemented at the agent level and assessed under realistic demographic and mobility constraints. Large-scale ABMs have been widely used during the COVID-19 pandemic to analyze intervention timing, healthcare demand, and compliance scenarios, thereby supporting public health decision-making [333, 334].

Despite their strengths, ABMs face several challenges. They are computationally intensive, especially for large-scale populations with fine spatial resolution. Furthermore, model calibration and validation require high-quality demographic, behavioral, and mobility data, which may be limited or uncertain. Increased model complexity can also reduce transparency and interpretability compared to simpler equation-based approaches. Nevertheless, advances in high-performance computing, parallel simulation methods, and data assimilation techniques continue to enhance the scalability and reliability of ABMs [335].

Overall, ABMs complement deterministic, stochastic, and network-based epidemic models by providing high-resolution, behaviorally realistic simulations. Their ability to integrate epidemiological processes with individual-level decision-making and social structure makes them an indispensable tool for modern epidemic forecasting and policy evaluation.

### Birth–death processes in epidemic modeling

Birth–death processes form a foundational class of stochastic epidemic models, where infection corresponds to the “birth” of an infective and recovery to its “death”. These models are particularly suitable for surveillance-based inference because they naturally align with reported case counts and event-driven transitions.

#### Population vs. density scaling

Arharas et al. [336] extended the classical Kurtz scaling [337–341] by defining spatially scaled birth–death processes in terms of densities per unit area. This formalism improves the interpretability of reproduction numbers across spatial resolutions and facilitates coupling with mobility or spatial surveillance data.

#### Birth–death processes with immigration

Bouzalmat et al. [342] proposed a linear birth–death process with immigration (LBDI) to represent exogenous infection seeding:

$$\begin{aligned}Q(i,i+1)&=\lambda i+\nu,\quad Q(i,i-1)\cr&=\mu i,\quad Q(i,i)=-(\lambda+\mu)i-\nu,\end{aligned}$$

where $\lambda$ denotes the infection rate, $\mu$ the recovery rate, and $\nu$ the immigration rate.

Such models are especially relevant for border screening, travel-related importation risk, and early outbreak monitoring.

#### Applications

Birth–death formulations have been applied to diseases including typhoid fever, cholera, and measles [343–346], facilitating likelihood-based parameter estimation and improving short-term forecasting performance [347].

### Qualitative properties and Public health implications

#### Noise-adjusted thresholds

In deterministic models, the basic reproduction number $\mathcal{R}_0$ defines the boundary between extinction and persistence. In stochastic settings, noise-induced fluctuations effectively reduce growth rates and may induce extinction even when deterministic thresholds predict persistence [42, 322, 323, 325]. This highlights the importance of probabilistic rather than purely deterministic intervention targets.

#### Extinction and persistence

Extinction can be established using supermartingale arguments applied to $\log I(t)$, while persistence follows from irreducibility and Lyapunov drift conditions. The existence of invariant measures [324, 348–350] enables long-term risk assessment and stability analysis of endemic equilibria under uncertainty.

#### Stationary distributions and ergodicity

Stationary distributions provide probabilistic descriptions of long-term prevalence levels and outbreak variability. Their existence relies on Lyapunov stability and Harris recurrence conditions, while jump–diffusion models require additional boundedness constraints [329, 351]. From a policy standpoint, these results support probabilistic burden forecasting and healthcare capacity planning.

Overall, while stochastic epidemic models are mathematically sophisticated, their integration with surveillance data, uncertainty quantification, and scenario analysis makes them indispensable tools for modern public health decision support, particularly in settings characterized by incomplete data, rapid behavioral change, and emerging pathogens.

### Numerical schemes and computation

Standard Euler–Maruyama and Milstein discretizations naturally extend to jump–diffusion models through compensated Poisson integrals. Numerical stability and positivity preservation require careful step-size selection to capture large jumps accurately and to avoid negative compartment counts [352–354].

**Fig. 5:**
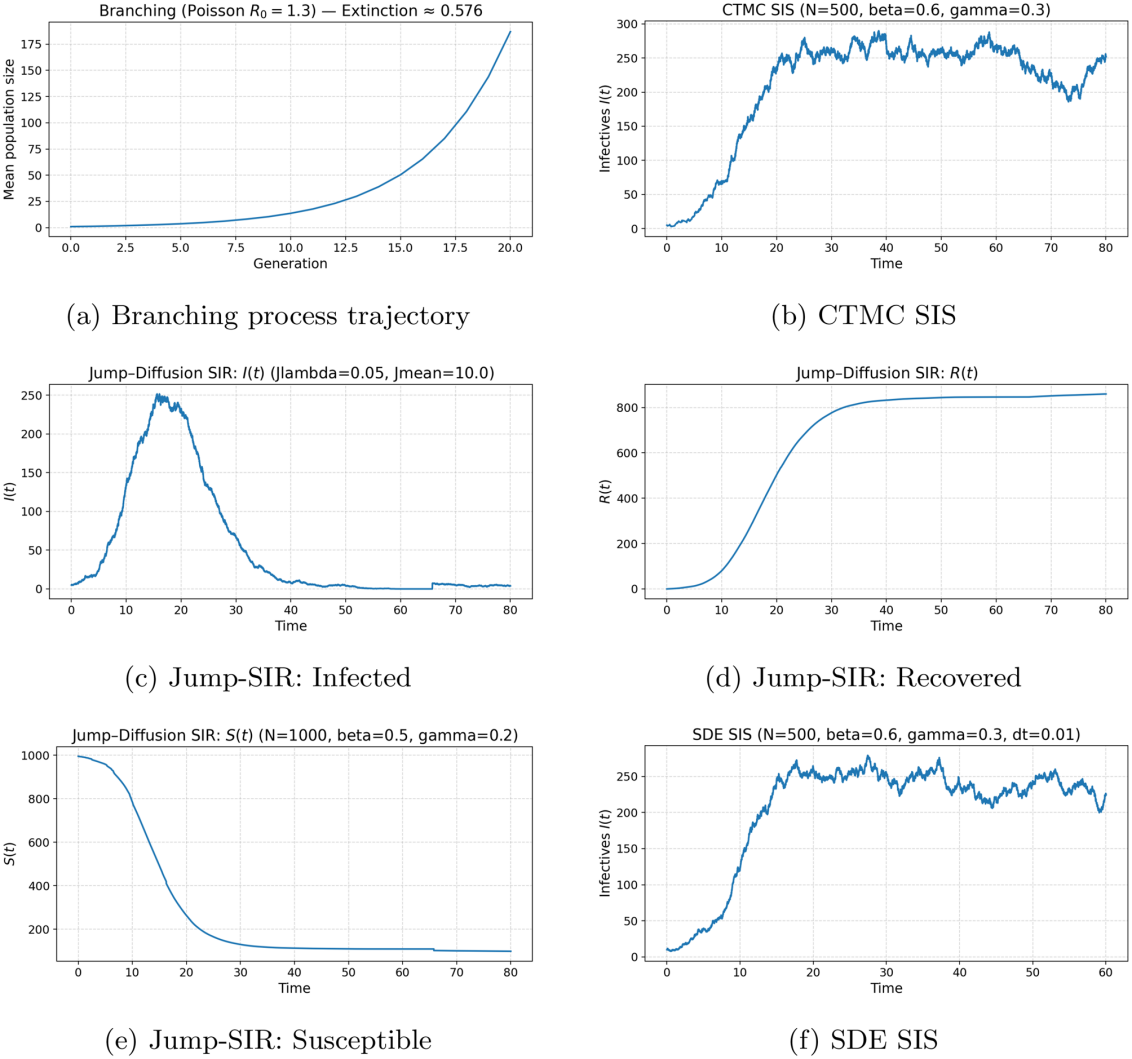
Stochastic epidemic model simulations: (**a**) branching process, (**b**) CTMC sis, (**c**) jump-diffusion sir infected, (**d**) jump-diffusion sir recovered, (**e**) jump-diffusion sir susceptible, and (**f**) sde sis

Figure 5 summarizes the simulation results obtained from the stochastic epidemic models considered in this study. Figure 5a shows the mean trajectory of the Galton–Watson branching process, highlighting the rapid extinction behavior that is characteristic of early-stage outbreak dynamics. The CTMC SIS simulation displayed in Figure 5b captures the discrete jump dynamics of the infectious population $I(t)$, generated using the Gillespie algorithm, and emphasizes the impact of demographic stochasticity.

Figures 5c–5e illustrate the jump–diffusion SIR model, where rare super-spreading events lead to abrupt increases in the infected population (Figure 5c), corresponding declines in the susceptible population (Figure 5e), and subsequent growth in the recovered compartment (Figure 5d). Finally, Figure 5f presents the diffusion approximation of the SIS model simulated using the Euler–Maruyama scheme, revealing continuous stochastic fluctuations around the corresponding deterministic mean-field trajectory.

Taken together, these results demonstrate how branching processes, CTMCs, SDEs, and jump–diffusion frameworks capture complementary aspects of stochastic epidemic dynamics across different modeling scales.

### Applications, recent advances, and open directions

Stochastic epidemic models have been applied across a wide range of epidemiological contexts. They have been used to explain the stochastic extinction of measles in island populations [17], to assess outbreak risks during the COVID-19 pandemic [355], to study long-term persistence of HIV and HBV infections [356, 357], and to evaluate vaccination and public health intervention policies [358]. In addition, jump–diffusion frameworks have been employed to account for the volatility and irregular prevalence patterns observed in empirical surveillance data [330].

Recent methodological developments include:

(i) Analyses of *backward bifurcation* phenomena under stochastic perturbations [359],
(ii) The introduction of *hybrid jump–diffusion models* that combine continuous fluctuations with discrete shocks [329],
(iii) The use of *fractional-order epidemic equations* that guarantee positivity and provide better memory representation of disease dynamics [147, 360], and
(iv) Investigations of *regime-switching* and *seasonally heterogeneous* systems that admit stationary distributions and complex endemic behaviors [361].

Despite these advances, several open research challenges remain. Key directions include:

- Establishing rigorous *multiscale network limits* that connect microscopic individual-based dynamics to macroscopic SDE or PDE representations;
- Improving *identifiability and parameter estimation* for jump–diffusion models using noisy and incomplete surveillance data;
- Developing *structure-preserving numerical schemes* that ensure positivity, stability, and accurate handling of rare jump events; and
- Designing *stochastic optimal control* frameworks that can manage interventions under deep model and data uncertainty.

Overall, stochastic epidemic models—spanning DTMCs, CTMCs, SDEs, jump–diffusions, branching processes, and birth–death formulations—offer refined epidemic thresholds that go beyond the deterministic $\mathcal{R}_0$, provide rigorous insights into extinction and persistence dynamics, and yield invariant laws that capture long-run stochastic stability. They continue to serve as indispensable tools in modern epidemiology, where variability, rare events, and randomness are inherent features of infectious disease dynamics.

## Network-based epidemic models

The study of networks has deep foundations in both the social sciences [362–364] and graph theory [365–367]. In epidemiology, networks describe how *hosts* interact through *contacts*; in social sciences, these are typically framed as *actors* and *relations*; while in graph theory, they appear as *nodes* and *edges*. Despite differences in terminology, all frameworks share a common goal—to characterize the structure of interactions within populations that mediate the spread of infectious diseases.

Formally, a contact network is represented as a graph $G = (V, E)$, where $V = \{1, 2, \ldots, N\}$ denotes the set of individuals (nodes), and $E \subseteq V \times V$ the set of epidemiologically relevant contacts (edges). An edge $(i,j) \in E$ indicates that infection can potentially be transmitted between individuals $i$ and $j$. The *degree* of node $i$, denoted $k_i$, quantifies the number of direct contacts and is given by

$$k_i = |\mathcal{N}_i| = \sum_{j=1}^{N} A_{ij},$$

where $A_{ij}$ is the adjacency matrix element ($A_{ij}=1$ if $(i,j)\in E$, and $A_{ij}=0$ otherwise).

Network-based epidemic models generalize the classical *homogeneous-mixing* assumption by explicitly incorporating contact heterogeneity. Instead of assuming that all individuals interact equally, the *mixing network* restricts possible transmission pathways to those defined by the network topology. This explicit representation of contact structure bridges microscopic, individual-level interactions with macroscopic epidemic dynamics, enabling the study of how heterogeneity and topology influence outbreak size, epidemic thresholds, and disease persistence [4, 368, 369].

**Classification of Network Models** Epidemic models on networks can be broadly classified into three main categories:

- *Static networks:* The contact structure remains fixed in time, representing stable relationships such as households, schools, or workplaces.
- *Dynamic networks:* Edges evolve over time to capture behavioral changes, mobility, or temporally varying contact patterns.
- *Data-driven networks:* Recent developments employ data science and machine learning techniques—particularly Graph Neural Networks (GNNs)—to infer latent network structures and to model epidemic dynamics directly from empirical or observational data.

These categories reflect an evolution from static abstractions to time-varying and data-adaptive representations, paralleling the increasing complexity and realism of modern epidemiological modeling.

### Static networks

Static network models assume a fixed contact structure throughout an epidemic episode. Analytical techniques such as heterogeneous mean-field (HMF) theory, pair approximation, and generating function methods reveal how network topology affects epidemic thresholds, outbreak sizes, and steady-state prevalence [368, 369].

At the microscopic level, for node $i$, the probability of infection in a short interval $\Delta t$ is given by

$$P(S_i \to I_i) = 1 - \exp\Big(-\beta \Delta t \sum_{j} A_{ij}\, 1_{\{x_j = I\}}\Big),$$

where $\beta$ is the per-contact transmission rate and $1_{\{x_j=I\}}$ indicates infection in neighbor $j$. Recovery occurs with probability

$$P(I_i \to S_i) \approx \gamma \Delta t,$$

where $\gamma$ is the recovery rate. Together, these expressions define the stochastic foundations of SIS and SIR processes on networks.

#### Heterogeneous mean-field (HMF) approximation

To capture degree heterogeneity, let $i_k(t)$ denote the fraction of infected nodes with degree $k$. The HMF equations [4, 370, 371] are

29 $$\frac{d i_k}{dt} = -\gamma i_k(t) + \beta k \big(1 - i_k(t)\big) \Theta(t), $$

30 $$\Theta(t) = \sum_{k\prime} \frac{k\prime P(k\prime)}{\langle k \rangle} i_{k\prime}(t),$$

where $\Theta(t)$ is the probability that a randomly chosen neighbor is infected. This framework reveals the disproportionate influence of highly connected nodes (“hubs”) on epidemic amplification and persistence.

#### Epidemic thresholds in networks

A key insight from network epidemiology is that the epidemic threshold depends on network topology. In HMF theory, the critical transmission probability $\beta_c$ satisfies

31 $$\beta_c = \frac{\langle k \rangle}{\langle k^2 \rangle},$$

where

$$\langle k \rangle = \sum_k k P(k), \quad \langle k^2 \rangle = \sum_k k^2 P(k).$$

Important implications follow:

- *Scale-free networks:* In networks with heavy-tailed degree distributions ($2 < \gamma \leq 3$), the second moment diverges ($\langle k^2 \rangle \to \infty$), leading to a vanishing epidemic threshold ($\beta_c \to 0$). Consequently, even extremely small transmission rates can sustain epidemics, highlighting the inherent structural vulnerability of such networks.
- *Analytical tractability:* HMF models provide a direct link between degree distribution and epidemic outcome, supporting fast analytic insight.
- *Limitations:* Real networks exhibit correlations, clustering, and community modularity, which shift epidemic thresholds relative to HMF predictions. Such corrections are crucial for realistic modeling.

**Fig. 6:**
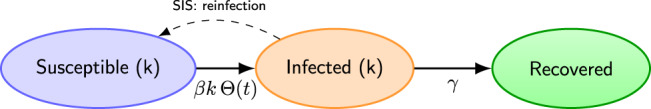
Node-level epidemic transitions in heterogeneous mean-field theory. Infection increases with degree $k$, while recovery is degree-independent. Coupling occurs through $\Theta(t)$, the probability that a random neighbor is infected

The node-level infection and recovery processes underlying the heterogeneous mean-field formulation are depicted in Fig. 6, highlighting the role of degree-dependent infection rates and the global coupling through the mean-field term $\Theta(t)$.

### Dynamic and temporal networks

In real populations, contacts are seldom static. *Dynamic network models* capture the evolution of social ties, mobility patterns, and behavioral adaptation over time. In the *activity-driven model* [372], each node $i$ becomes active with probability $a_i$ and establishes $m$ transient connections that dissolve shortly thereafter. The corresponding SIR dynamics can be expressed as

$$\frac{d I_a}{dt} = -\gamma I_a + \beta a S_a \Theta(t) + \beta S_a \langle a\prime I_{a\prime} \rangle,$$

where the first term represents recovery, the second describes infections generated by the activity of node $i$, and the last term accounts for infections arising from active neighbors with activity level $a\prime$.

*Temporal network analysis* extends this framework by incorporating empirical time-resolved contact data. Studies have shown that bursty, clustered, or highly heterogeneous interaction patterns can substantially slow epidemic spreading compared to memoryless (Poisson) contact processes [373–376]. Such models thus provide a crucial bridge between theoretical constructs and real-world temporal heterogeneity observed in proximity, mobility, and digital contact datasets.

### Graph neural network (gnn) models

Recent developments in artificial intelligence have introduced graph neural networks (GNNs) as a powerful unifying framework between data-driven and mechanistic modeling. GNNs represent individuals as nodes and their interactions as edges, learning both local and global dependencies through message passing [377]. Node embeddings $h_i^{(\ell)}$ evolve as

$$h_i^{(\ell+1)} = \sigma\Big(W^{(\ell)} h_i^{(\ell)} + \sum_{j \in \mathcal{N}(i)} \phi(h_i^{(\ell)},h_j^{(\ell)},A_{ij}) \Big),$$

where $W^{(\ell)}$ are learnable weights, $\phi(\cdot)$ aggregates neighbor information, and $\sigma(\cdot)$ is a nonlinear activation.

Applications include outbreak detection, hotspot surveillance, incidence forecasting, and policy evaluation [378–381]. Two complementary directions have emerged:

- *Purely neural models:* Spatio-temporal GNNs that learn epidemic dynamics directly from data, without explicit mechanistic priors.
- *Hybrid models:* Architectures embedding compartmental dynamics (e.g., SIR, SEIR) within GNN layers, achieving interpretability while preserving consistency with epidemiological theory [380, 381].

GNN-based epidemic models thus offer scalable and adaptive tools that integrate graph structure, temporal dynamics, and heterogeneous data sources within a single learning framework.

### Simulation examples

To complement the theoretical exposition, illustrative simulations demonstrate how network structure influences epidemic persistence and spread.

#### Sis dynamics on an Erdős–rényi network

**Fig. 7:**
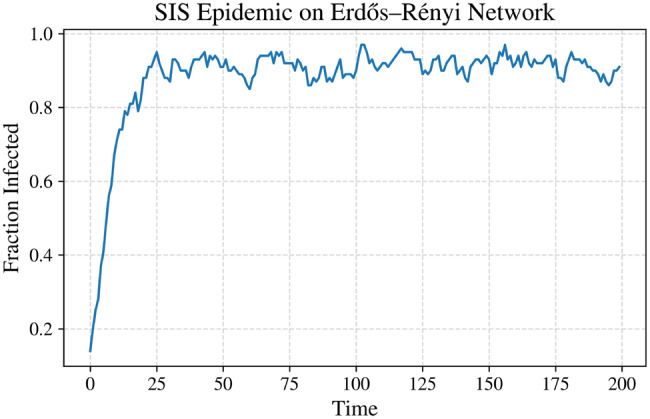
Illustrative sis epidemic simulation on a static erdős–rényi network ($N=100$, $\langle k \rangle=6$). The infection persists at endemic levels when $\beta / \gamma > \beta_c^{-1}$

We begin by examining a susceptible–infected–susceptible (SIS) process on an Erdős–Rényi (ER) network with $N = 100$ nodes and an average degree of $\langle k \rangle = 6$. Using an infection rate $\beta = 0.05$, a recovery rate $\gamma = 0.02$, and an initial infection level of $10\%$, the temporal evolution of the infected fraction is shown in Fig. 7. The results indicate that the infection persists at endemic levels when $\beta / \gamma > \beta_c^{-1}$, consistent with mean-field theoretical predictions.

#### Sir dynamics on a random network

**Fig. 8:**
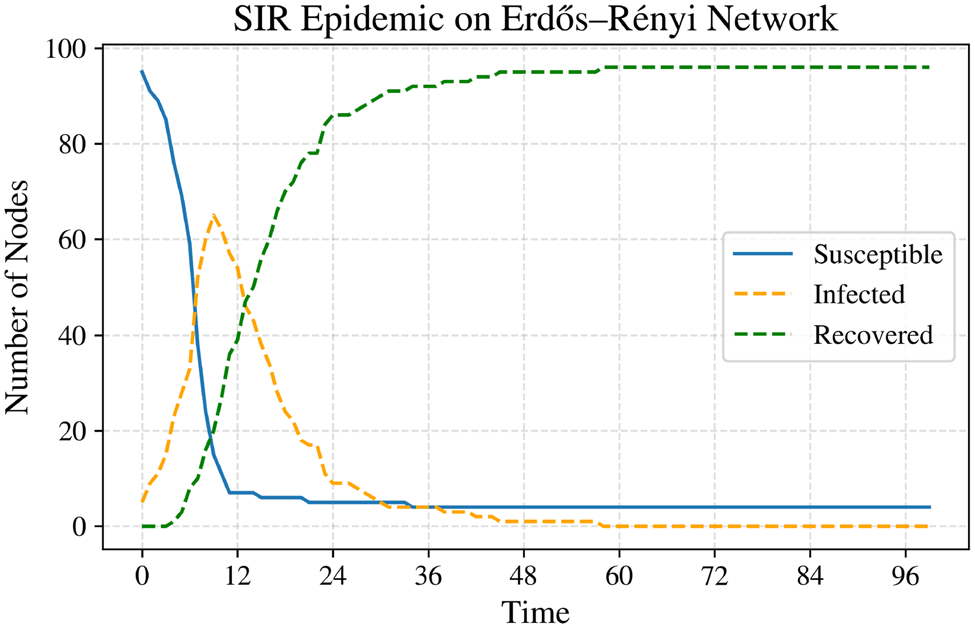
Sir epidemic simulation on a random network with $N = 100$ nodes. The susceptible (blue), infected (red), and recovered (green) populations are shown as functions of time. The simulation framework can be readily adapted for the sis model or for alternative network topologies

A similar setup can be extended to the susceptible–infected–recovered (SIR) model. Here, an Erdös–Rényi random graph with $N = 100$ nodes and connection probability $p = 0.05$ is simulated using a discrete-time Python implementation. Transmission occurs at rate $\beta = 0.2$ and recovery at $\gamma = 0.1$. The resulting epidemic curves, shown in Fig. 8, illustrate the temporal evolution of the SIR compartments and demonstrate how network topology affects epidemic peak and duration.

### Comparison between network-based models and structured compartmental models

Network-based epidemic models differ fundamentally from traditional structured compartmental models in how population interactions are represented. While structured compartmental models (such as age-structured, spatially structured, or metapopulation SEIR frameworks) group individuals into aggregated subpopulations and assume homogeneous mixing within each subgroup, network-based models explicitly represent individuals as nodes and epidemiologically relevant contacts as edges. This explicit representation preserves the heterogeneity of contact patterns, including degree variability, clustering, community structure, and hub formation, which are often averaged out in compartmental formulations.

In network-based models, disease transmission occurs along contact links rather than through population-level mass-action terms. This allows direct incorporation of realistic social contact structures and enables the study of how network topology influences epidemic thresholds, outbreak sizes, and persistence. In contrast, structured compartmental models rely on predefined mixing matrices or spatial coupling terms to approximate interaction patterns, which can oversimplify real-world contact heterogeneity.

Moreover, network models naturally support the analysis of targeted intervention strategies such as hub vaccination, contact-based quarantine, and contact tracing, which are difficult to implement accurately in aggregated frameworks. However, compartmental models remain computationally efficient and analytically tractable, making them suitable for large-scale forecasting and theoretical analysis. Consequently, network-based and structured compartmental models should be viewed as complementary approaches: the former offers high-resolution representation of contact heterogeneity, while the latter provides scalable and interpretable population-level insights.

### Applications and case studies

Network-based epidemic models have been widely applied to diverse infectious diseases, providing mechanistic insight into how contact structures influence transmission dynamics. In static networks, studies of HIV and influenza have demonstrated that highly connected individuals act as super-spreaders, informing the design of targeted intervention strategies [382–384]. Dynamic network models that incorporate mobility patterns and temporal contact data have further elucidated how human movement and behavioral adaptation shape epidemic waves, as observed during the COVID-19 and Ebola outbreaks [385, 386]. More recently, data-driven and AI-enhanced approaches—particularly those employing graph neural networks (GNNs)—have enabled accurate epidemic forecasting and hotspot detection by integrating demographic, behavioral, and mobility information [387, 388].

From analytical heterogeneous mean-field (HMF) formulations to AI-augmented graph learning frameworks, network-based models provide a unified approach to studying contagion processes in structured populations. They reveal how heterogeneity, temporal evolution, and adaptive connectivity jointly influence epidemic thresholds, persistence, and control strategies, thereby advancing both predictive and prescriptive epidemiology.

## Data-driven and AI-based epidemic models

Classical mechanistic models—such as SIR, SEIR, or network-based frameworks—encode domain knowledge through explicitly defined compartments, parameters, and transmission functions. While they have long provided interpretable insights into disease dynamics, their predictive power often declines in fast-evolving contexts characterized by reporting noise, behavioral feedback, and spatial heterogeneity. The exponential growth of epidemiological, mobility, and behavioral data has therefore motivated a paradigm shift toward *data-driven modeling*, which learns patterns directly from observations rather than fixed mechanistic assumptions [389–391]. This section surveys key components of the modern data–AI epidemic modeling pipeline, highlighting the continuum between statistical inference and deep learning, and their hybridization with mechanistic theory.

### Data modalities and modeling pipeline

Contemporary epidemic analytics integrate multimodal data streams spanning different spatial, temporal, and behavioral scales [392, 393]. Typical pipelines involve:

- **Epidemiological signals:** case counts, hospitalizations, deaths, and testing data;
- **Behavioral and mobility indicators:** mobility from mobile devices, policy stringency indices, social media sentiment;
- **Environmental covariates:** temperature, humidity, and pollution levels influencing pathogen viability;
- **Clinical data:** patient-level features, comorbidities, and vaccination status.

Before modeling, extensive pre-processing is essential: de-noising and imputation mitigate reporting gaps, *nowcasting* corrects for delays, and adjustments account for holiday or surveillance effects. Feature engineering then extracts informative temporal patterns (lags, rolling averages, renewal kernels), seasonal and weather indicators, or policy embeddings. Finally, learning and inference proceed via statistical or machine learning models, while evaluation relies on rolling-origin validation, calibration, and probabilistic scoring (e.g., CRPS or log-score). This pipeline provides the structural backbone for both statistical and AI-based forecasting systems.

### Data-driven statistical models

Statistical approaches form the bridge between mechanistic and machine learning paradigms. They infer latent epidemiological quantities from data under explicit uncertainty assumptions [389, 390].

- **Generalized linear and state-space models** estimate incidence, growth rates, or effective reproduction numbers $R_t$ using Bayesian filtering or smoothing.
- **Renewal models** capture infection chains via generation-interval weights $w_s$, where daily incidence $I_t$ follows
  $$ I_t \sim \mathrm{Pois}\!\left(R_t \sum_{s=1}^{t-1} w_s I_{t-s}\right), \qquad R_t \leftarrow \text{stochastic process}, $$
  enabling near–real-time inference of $R_t$ and epidemic momentum.
- **Hierarchical and multilevel models** pool information across regions, allowing for both global and local effects (e.g., mobility, interventions, and healthcare capacity).

Such models remain the workhorse for short-term situational awareness and probabilistic scenario analysis, where interpretability and uncertainty quantification are critical.

### Machine learning and ai models

While statistical models emphasize transparency, machine learning excels at capturing high-dimensional nonlinear relationships. These methods leverage multimodal data to learn complex spatio-temporal dependencies [391, 394–399].

- **Classical ML** methods—SVMs, decision trees, ensembles, and Naïve Bayes—are used for outbreak classification, hotspot detection, and early warning.
- **Deep learning** architectures exploit spatial and temporal hierarchies: CNNs extract spatial correlations, RNNs/LSTMs model temporal evolution, and Transformers handle long-range dependencies and multi-horizon forecasts. For example in [400] an investigation machine learning and deep learning approaches for forecasting malaria incidence in the Adamaoua region of Cameroon, highlighting the importance of data-driven predictive models in epidemic monitoring and public health planning has been carried out.
- **Graph neural networks (GNNs)** integrate epidemiological networks and mobility graphs, learning joint space–time transmission patterns.
- **Training regimes** include self-/semi-supervised learning and transfer or zero-shot adaptation for data-scarce scenarios.

Collectively, these models have improved short- and medium-term forecasts, digital surveillance, and individualized risk stratification [355, 401–404].

### Hybrid mechanistic–AI integration

Recent advances in artificial intelligence (AI) have accelerated the integration of data-driven learning with mechanistic epidemiological models, forming a new class of hybrid frameworks that aim to combine interpretability, biological realism, and predictive flexibility [405]. These integrated approaches address fundamental limitations of purely mechanistic models, such as parameter uncertainty, rigid functional assumptions, and limited capacity to assimilate heterogeneous data sources, while avoiding the poor extrapolation and limited causal interpretability of purely data-driven models.

Following recent comprehensive reviews, hybrid mechanistic–AI approaches can be broadly categorized into several methodological paradigms [405]. First, *mechanism-informed machine learning models* incorporate epidemiological quantities such as reproduction numbers, mobility-adjusted contact rates, compartmental states, or renewal-equation outputs as structured inputs or constraints for neural networks and tree-based models. This strategy improves forecasting performance while preserving epidemiological consistency [406].

Second, *neural-augmented mechanistic models* embed learnable components directly into epidemiological equations. Physics-informed neural networks and epidemiology-informed neural networks enforce compartmental dynamics through loss functions while simultaneously learning unknown parameters, transmission rates, or latent forcing terms from data [406, 407]. This approach enables end-to-end training while maintaining explicit disease transmission structure and has demonstrated improved identifiability and robustness under sparse or noisy observations.

Third, *surrogate modeling and neural emulation* replace computationally expensive simulators, particularly agent-based and spatially explicit models, with lightweight neural approximations. These surrogates enable efficient Bayesian calibration, uncertainty quantification, and rapid scenario evaluation [405, 408]. This paradigm is especially important for real-time policy analysis, where repeated simulation is computationally prohibitive.

Fourth, *AI-driven intervention optimization frameworks* integrate reinforcement learning and optimal control with mechanistic disease models. In these approaches, the epidemiological simulator acts as an environment in which AI agents learn adaptive non-pharmaceutical intervention strategies, vaccination policies, or mobility restrictions under dynamic transmission conditions [409]. Such methods allow explicit optimization under realistic epidemic constraints and resource limitations.

Finally, *synthetically trained and theory-guided AI models* leverage large datasets generated from mechanistic simulators to train neural networks capable of learning transmission dynamics on networks or spatial systems [379, 410]. These approaches help overcome data scarcity and enhance generalization across epidemiological scenarios.

Despite their promise, hybrid frameworks face important challenges, including model transparency, robustness to dataset bias, limited availability of high-resolution surveillance data, and the need for systematic benchmarking against traditional mechanistic and statistical models [405]. Nevertheless, the growing availability of multimodal epidemiological data and advances in constrained learning, uncertainty-aware neural networks, and high-performance computing are rapidly expanding the practical utility of integrated models.

Overall, hybrid mechanistic–AI models represent a critical step toward scalable, interpretable, and data-adaptive epidemic modeling. By unifying causal disease dynamics with modern machine learning, these frameworks provide a powerful foundation for real-time forecasting, policy optimization, and scenario-based public health decision support.

### Evaluation, uncertainty, and deployment

For operational deployment, rigorous evaluation is indispensable. Robust pipelines involve:

- **Backtesting** via rolling-origin forecasts with embargo windows to avoid information leakage;
- **Probabilistic accuracy** through calibration curves and proper scoring rules (e.g., log-score, CRPS);
- **Drift monitoring** to detect data shifts, backfill revisions, or changing reporting regimes;
- **Decision support** aligning metrics with intervention costs, triage thresholds, and surge planning.

Integrating uncertainty quantification—via Bayesian deep learning, ensembles, or conformal prediction—remains key for trustworthy policy applications.

### Strengths and limitations of ai approaches

#### Advantages and limitations

AI-based epidemic models offer substantial advantages, including high *flexibility* in learning complex nonlinear transmission patterns with minimal structural assumptions, the ability to perform *multimodal data fusion* across heterogeneous sources (e.g., case counts, mobility traces, medical images, and social media signals), and strong *adaptivity* through rapid retraining as new surveillance data become available. These features make AI-driven approaches particularly attractive for short-term forecasting, real-time outbreak monitoring, and large-scale data integration tasks.

However, despite their promising predictive performance, AI- and machine-learning-based epidemic models face several important limitations that must be carefully considered in applied public health settings. Their primary weaknesses stem from strong *data dependence*, where limited data quality, incomplete reporting, and uneven geographic coverage can substantially degrade performance and amplify bias [411]. A central challenge is *interpretability*: deep neural networks and graph-based architectures often operate as black-box models, making it difficult to extract mechanistic insight or to justify policy decisions to public health authorities and stakeholders [412]. Moreover, most data-driven approaches are fundamentally correlational and do not explicitly encode causal transmission mechanisms, which limits their reliability for counterfactual analysis and intervention evaluation [413].

Additional challenges include *overfitting*, particularly when training datasets are restricted to short outbreak windows or affected by reporting delays and under-ascertainment, as well as *data leakage*, where temporal or spatial information inadvertently enters training pipelines and leads to overly optimistic performance estimates that fail to generalize to real-time forecasting scenarios [414]. Furthermore, AI models trained on data from specific regions, demographic structures, or outbreak phases often exhibit limited *transferability* when deployed in new geographic contexts or during emerging epidemics involving novel pathogens [415].

Together, these limitations highlight the importance of rigorous validation protocols, uncertainty quantification, transparent reporting standards, and the adoption of hybrid modeling strategies that integrate mechanistic epidemiological structure with data-driven learning [416]. Such hybrid frameworks provide a promising pathway to combine predictive accuracy with interpretability, robustness, and policy relevance, thereby enhancing the reliability of AI-assisted epidemic decision support systems.

### Future directions

AI and data-driven methods now complement mechanistic epidemiology by enabling adaptive forecasting and real-time response [391–393]. Future progress lies in:

- **Tighter hybridization:** integrating physics- or epidemiology-informed architectures, constrained learning (positivity, conservation), and causal graph discovery;
- **Low-data regimes:** foundation or large pre-trained models adapted via self-/few-shot learning for emergent pathogens;
- **Causal inference:** rigorous counterfactual evaluation with identifiability and calibrated uncertainty;
- **Governance and access:** reproducible, privacy-preserving, and equitable data infrastructures;
- **Operational AI assistants:** domain-specialized “AI–infectious disease” systems combining mechanistic simulators with explainable ML for decision support.

Data-driven and AI-based modeling expands the epidemiological toolkit. When fused with mechanistic reasoning and rigorous uncertainty quantification, these methods enable actionable, explainable, and equitable epidemic intelligence [402–404, 417].

## Comparative analysis of modeling approaches

Different infectious disease modeling frameworks exhibit complementary strengths and limitations, making their suitability highly context-dependent. Deterministic compartmental models, such as SIR and SEIR, offer analytical tractability and low computational cost, allowing rapid scenario exploration and estimation of epidemiological thresholds such as the basic reproduction number. However, their assumption of homogeneous mixing limits their ability to capture individual-level heterogeneity and stochastic fluctuations, particularly during early outbreak phases.

In contrast, stochastic models explicitly incorporate randomness in transmission and recovery processes, making them more suitable for small populations and early epidemic dynamics. While they provide improved uncertainty quantification, their higher computational demands and reduced analytical interpretability can limit large-scale deployment.

Network-based models address the limitations of homogeneous mixing by explicitly representing contact structures. These models are particularly effective in studying targeted interventions, such as vaccination of highly connected individuals. Nevertheless, they require detailed contact data, which is often unavailable or incomplete in real-world settings.

Spatial and PDE-based models enable the investigation of geographic heterogeneity and mobility-driven transmission patterns. They are valuable for evaluating region-specific interventions but require extensive parameterization and computational resources.

Finally, data-driven and AI-based approaches offer strong predictive capabilities when large datasets are available. However, their limited interpretability and dependence on data quality highlight the importance of combining them with mechanistic models to ensure epidemiological consistency and policy relevance.

Overall, hybrid frameworks that integrate mechanistic modeling with data-driven methods appear most promising for balancing interpretability, predictive accuracy, and real-world applicability.

In addition to the descriptive overview of modeling paradigms, the comparative analysis in Table 1 highlights key trade-offs that guide the selection of appropriate frameworks for specific epidemiological contexts. Deterministic compartmental models remain attractive for rapid scenario analysis and policy communication due to their interpretability and low computational cost, but their simplifying assumptions limit realism in heterogeneous populations. In contrast, stochastic and network-based models better capture individual-level variability, superspreading events, and contact heterogeneity, making them more suitable for early outbreak detection and targeted intervention design, albeit at the cost of increased computational complexity and data requirements. Spatial PDE-based models provide unique insight into geographic spread and mobility-driven transmission patterns, which is critical for regional containment strategies and resource allocation. Finally, data-driven and AI-enhanced approaches offer strong predictive performance and real-time adaptability when large datasets are available; however, their limited interpretability and dependence on data quality pose challenges for transparent policy use. Together, this synthesis underscores that no single modeling framework is universally optimal, and hybrid strategies that combine mechanistic interpretability with data-driven adaptability increasingly represent best practice for modern epidemic forecasting and decision support.

**Table 1:** Comparison of major infectious disease modeling approaches, highlighting their advantages, limitations, and typical application scenarios

Model Type	Key Advantages	Main Limitations	Typical Use-Cases
Deterministic compartmental models (SIR, SEIR)	Simple structure, analytical tractability, low computational cost, interpretable epidemiological parameters (e.g., $R_0$)	Assume homogeneous mixing, ignore stochastic effects, limited representation of individual heterogeneity	Large-scale epidemic trends, rapid scenario analysis, evaluation of broad public health interventions
Stochastic models	Capture randomness and uncertainty, realistic early outbreak dynamics, extinction probability estimation	Higher computational cost, reduced analytical simplicity, parameter estimation can be challenging	Small populations, outbreak risk assessment, uncertainty quantification, early epidemic phase analysis
Network-based models	Explicit representation of contact heterogeneity, suitable for targeted intervention strategies, realistic transmission pathways	Require detailed contact data, increased model complexity, scalability challenges for large networks	Contact tracing analysis, targeted vaccination strategies, social network-driven transmission studies
Spatial and PDE-based models	Incorporate geographic heterogeneity and mobility, capture spatial spread patterns and wave propagation	Computationally intensive, complex parameterization, higher data requirements	Regional outbreak analysis, mobility restrictions, spatial intervention planning
Data-driven and AI-based models	High predictive capability with large datasets, adaptable to real-time data streams, automated pattern extraction	Limited interpretability, dependence on data quality and availability, potential lack of epidemiological transparency	Short-term forecasting, real-time surveillance, anomaly detection, rapid outbreak monitoring
Hybrid and integrated models	Combine mechanistic interpretability with data-driven accuracy, improved robustness and flexibility	Increased implementation complexity, higher computational and data demands	Decision support systems, policy evaluation, real-time epidemic management

### Model selection, parameter estimation, and validation using real-world data

Effective application of infectious disease models in public health practice requires careful consideration of model selection, robust parameter estimation, and systematic validation against real-world data. While theoretical models provide valuable insights into disease dynamics, their practical utility depends on how well they are aligned with available data, epidemiological context, and decision-making objectives.

*Model selection* represents the first critical step in applied epidemic modeling. The choice of modeling framework should be guided by the scale of analysis, data availability, computational resources, and the specific policy or research question. Simple compartmental models such as SIR and SEIR are often preferred for rapid assessment and large-scale forecasting due to their interpretability and computational efficiency. However, when heterogeneity in contact patterns, spatial mobility, or demographic structure plays a significant role, network-based, spatially explicit, or agent-based models may provide more realistic representations. In practice, a balance must be achieved between model complexity and data support, as overly complex models may suffer from parameter identifiability issues and increased uncertainty when data are limited [3].

*Parameter estimation* is central to transforming conceptual models into predictive tools. Epidemiological parameters such as transmission rates, recovery rates, incubation periods, and intervention effects are typically inferred from surveillance data, hospitalization records, serological studies, and mobility datasets. Common estimation approaches include least-squares optimization, maximum likelihood estimation, and Bayesian inference. Bayesian methods are increasingly adopted because they allow the incorporation of prior knowledge, explicit representation of uncertainty, and probabilistic forecasting. Data assimilation techniques, such as ensemble Kalman filtering and particle filtering, have further enabled real-time model updating by continuously integrating new observations. These approaches are particularly valuable during rapidly evolving outbreaks, where parameters may change over time due to behavioral adaptation, public health interventions, or pathogen evolution [17].

*Model validation* is essential to assess predictive performance and ensure reliability for policy guidance. Validation strategies include retrospective fitting and hindcasting, where models are calibrated using historical data and evaluated on their ability to reproduce observed epidemic trajectories. Cross-validation using independent datasets or regional subsets provides additional robustness checks. Furthermore, comparison of predicted outcomes—such as case counts, hospitalization rates, and peak timing—with observed data helps identify systematic biases and structural limitations. Sensitivity analysis plays a complementary role by quantifying how uncertainty in parameters propagates to model outputs, thereby highlighting influential parameters and critical assumptions.

In addition to traditional validation approaches, *uncertainty quantification and scenario analysis* have become integral components of applied epidemic modeling. Uncertainty intervals, ensemble forecasts, and probabilistic projections provide policymakers with a range of plausible outcomes rather than single-point predictions. Scenario-based modeling enables evaluation of alternative intervention strategies, such as vaccination coverage levels, social distancing measures, and travel restrictions, under different assumptions about behavioral compliance and epidemic severity.

The increasing availability of *real-world data streams* has significantly enhanced the calibration and validation of epidemic models. These data include high-resolution mobility data, genomic surveillance, wastewater monitoring, digital contact tracing records, and real-time reporting systems. Integrating such heterogeneous data sources improves model realism and supports early outbreak detection and adaptive intervention planning. However, data quality issues, reporting delays, under-ascertainment, and measurement noise remain major challenges. Addressing these limitations requires robust statistical methods, transparent reporting of uncertainty, and continuous model refinement [418].

Overall, effective integration of model selection, parameter estimation, and validation forms the foundation of reliable epidemic forecasting and evidence-based decision support. By combining mechanistic understanding with real-world data and rigorous evaluation frameworks, infectious disease models can better support public health preparedness, real-time response, and long-term policy planning.

## Challenges, conclusion and future directions

Despite significant progress in mathematical and computational epidemiology, several persistent challenges hinder the reliability and social impact of epidemic modeling. Data quality and availability remain major constraints, as surveillance systems often produce incomplete, delayed, or biased datasets due to underreporting, inconsistent testing, and limited demographic coverage. Integrating diverse sources such as clinical, mobility, and genomic data further complicates analysis and raises privacy concerns. Model uncertainty and parameter identifiability also pose critical issues, since different model structures or parameter combinations can yield similar epidemic trajectories, leading to ambiguity in inference and prediction. Quantifying and communicating these uncertainties through robust statistical frameworks, such as Bayesian inference and ensemble modeling, is essential to avoid overconfidence in projections. In addition, ethical and policy considerations play a central role: data-driven models must protect individual privacy, promote fairness, and account for social disparities in healthcare access and intervention outcomes. Ensuring transparency, accountability, and equity in model-based decision-making therefore remains an open and interdisciplinary challenge, demanding closer collaboration among mathematicians, data scientists, public health experts, and policymakers.

Mathematical modeling continues to serve as a cornerstone of infectious disease research, offering critical insights into epidemic dynamics, intervention effectiveness, and public health planning. Over time, the field has evolved from classical compartmental models such as SIR and SEIR to a wide spectrum of advanced approaches, including stochastic, network-based, agent-based, and spatially explicit frameworks. Each class of models contributes distinct strengths: deterministic systems effectively describe large-scale epidemic trends, while stochastic and network formulations capture heterogeneity, randomness, and complex contact structures inherent in real populations.

The recent surge in data availability and computational capacity has accelerated the integration of data-driven and artificial intelligence (AI) methods with traditional mechanistic frameworks. Hybrid modeling paradigms that unite interpretability with predictive power show great promise for real-time forecasting, resource optimization, and evidence-based policymaking. Nevertheless, key challenges persist, including data scarcity during early outbreak phases, parameter uncertainty, the opacity of black-box AI systems, and the pressing need for interdisciplinary collaboration among mathematicians, epidemiologists, data scientists, and decision-makers.

Looking ahead, the most significant advances will likely emerge from a deeper synthesis of mechanistic and data-driven paradigms, underpinned by developments in high-performance computing, causal inference, and explainable AI. Strengthening the bridge between theoretical modeling and practical policy application will ensure that models remain both scientifically rigorous and operationally actionable. By uniting mathematical theory, computational innovation, and empirical insight, the future of infectious disease modeling holds the potential not only to enhance epidemic preparedness and response but also to contribute to the prevention of future global health crises.

### Comparative analysis of modeling approaches

Different infectious disease modeling frameworks exhibit complementary strengths and limitations, making their suitability highly context-dependent. Deterministic compartmental models, such as SIR and SEIR, offer analytical tractability and low computational cost, allowing rapid scenario exploration and estimation of epidemiological thresholds such as the basic reproduction number. However, their assumption of homogeneous mixing limits their ability to capture individual-level heterogeneity and stochastic fluctuations, particularly during early outbreak phases.

In contrast, stochastic models explicitly incorporate randomness in transmission and recovery processes, making them more suitable for small populations and early epidemic dynamics. While they provide improved uncertainty quantification, their higher computational demands and reduced analytical interpretability can limit large-scale deployment.

Network-based models address the limitations of homogeneous mixing by explicitly representing contact structures. These models are particularly effective in studying targeted interventions, such as vaccination of highly connected individuals. Nevertheless, they require detailed contact data, which is often unavailable or incomplete in real-world settings.

Spatial and PDE-based models enable the investigation of geographic heterogeneity and mobility-driven transmission patterns. They are valuable for evaluating region-specific interventions but require extensive parameterization and computational resources.

Finally, data-driven and AI-based approaches offer strong predictive capabilities when large datasets are available. However, their limited interpretability and dependence on data quality highlight the importance of combining them with mechanistic models to ensure epidemiological consistency and policy relevance.

Overall, hybrid frameworks that integrate mechanistic modeling with data-driven methods appear most promising for balancing interpretability, predictive accuracy, and real-world applicability.

## Data Availability

There is no data associated to this research article.

## Abbreviations

ABM: Agent-Based Model
AI: Artificial Intelligence
CTMC: Continuous-Time Markov Chain
DTMC: Discrete-Time Markov Chain
DFE: Disease-Free Equilibrium
GNN: Graph Neural Network
HMF: Heterogeneous Mean-Field
ODE: Ordinary Differential Equation
PDE: Partial Differential Equation
R$_0$: Basic Reproduction Number
SEIR: Susceptible–Exposed–Infected–Recovered
SEIRS: Susceptible–Exposed–Infected–Recovered–Susceptible
SIR: Susceptible–Infected–Recovered
SIRD: Susceptible–Infected–Recovered–Deceased
SIS: Susceptible–Infected–Susceptible
SDE: Stochastic Differential Equation
